# Identification and Optimization of a Truncated Hs‐1‐Derived Antimicrobial Peptide for Enhanced Broad‐Spectrum Antiviral Activity

**DOI:** 10.1002/cmdc.70380

**Published:** 2026-07-08

**Authors:** Carla Zannella, Annalisa Chianese, Rosa Giugliano, Roberta Della Marca, Alessandra Monti, Martina Dragone, Gaetano Malgieri, Nunzianna Doti, Carla Isernia, Anna De Filippis, Massimiliano Galdiero

**Affiliations:** ^1^ Department of Woman, Child and General and Specialized Surgery University of Campania “Luigi Vanvitelli” Naples Italy; ^2^ Department of Life Sciences, Health and Health Professions Link Campus University Rome Italy; ^3^ Department of Veterinary Medicine and Animal Production University of Naples Federico II Naples Italy; ^4^ Institute of Biostructures and Bioimaging (IBB) National Research Council (CNR) Naples Italy; ^5^ Department of Environmental, Biological, and Pharmaceutical Sciences and Technologies University of Campania “Luigi Vanvitelli” Caserta Italy; ^6^ Complex Operative Unit of Virology and Microbiology University Hospital of Campania “Luigi Vanvitelli” Naples Italy

**Keywords:** antimicrobial peptides (AMPs), antiviral activity, frog‐derived peptides, nuclear magnetic resonance (NMR), peptide optimization

## Abstract

Antimicrobial peptides (AMPs) represent promising scaffolds for the development of novel broad‐spectrum antiviral therapeutics. Based on structural modeling data, we applied a systematic sequence downsizing approach to the 20‐amino‐acid AMP Hs‐1 from *Hypsiboas semilineatus* to identify its minimal active region. A series of N‐ and C‐terminally truncated derivatives was synthesized to preserve the predicted amphipathic α‐helical core. Circular dichroism showed that the peptides adopt a random‐coil conformation in aqueous solution and transition to an α‐helical structure in hydrophobic environments, which is essential for membrane disruption. Antiviral screening against enveloped viruses revealed that the peptides interfere with the early stages of infection while maintaining a favorable safety profile. Notably, variant Hs‐1[7–20] showed a two‐to‐four‐fold potency enhancement over the parent peptide, indicating the successful removal of a detrimental N‐terminal segment. Further optimization via N‐terminal acetylation and C‐terminal amidation yielded Hs‐1[7–20]mod, which exhibited increased antiviral efficacy and serum stability, achieving low‐micromolar IC50 values (0.5–0.7 µM) under virus pretreatment conditions while maintaining low toxicity (CC50 > 100 µM). In conclusion, Hs‐1 was successfully optimized into a potent, stable, and safe antiviral candidate Hs‐1[7–20]mod.

## Graphical abstract

Can a shorter peptide be a better antiviral? Structure‐guided optimization of the amphibian peptide Hs‐1 reveals a minimal α‐helical core that, after terminal modification, displays enhanced stability and submicromolar broad‐spectrum activity against enveloped viruses while maintaining a favorable safety profile.

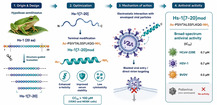

## 1 Introduction

Viral pathogen outbreaks continue to pose significant challenges to global health systems in the modern era. Over the past century, numerous devastating infectious disease outbreaks have emerged, predominantly caused by RNA viruses, including coronaviruses (CoVs) such as severe acute respiratory syndrome CoV 2 (SARS‐CoV‐2), flaviviruses such as West Nile virus (WNV) and dengue virus (DENV), and filoviruses such as Ebola virus (EBOV) [1, 2, 3, 4]. In addition, infections caused by influenza A virus, hepatitis B virus (HBV), hepatitis C virus (HCV), and human immunodeficiency virus (HIV) remain a persistent risk to human health [5, 6], highlighting the persistent challenges posed by viral pathogens worldwide and the urgent need for the development of new antiviral strategies. While viral replication mechanisms vary among viruses, the viral life cycle generally comprises several conserved stages, including entry, biosynthesis, assembly, and release. These shared processes often serve as strategic targets for antiviral development. Viral entry, the initial step of infection, involves multiple complex processes, including receptor recognition, surface protein priming, endocytosis, and membrane fusion. Accordingly, all components mediating viral entry constitute key therapeutic targets [7, 8, 9]. In this context, antimicrobial peptides (AMPs) have emerged as promising antiviral agents due to their ability to interfere with viral entry and other stages of the viral life cycle, offering a broad‐spectrum approach to combat diverse viral pathogens.

AMPs, a subset of natural host defense peptides, are typically small (≤50 amino acids), cationic, and amphiphilic molecules that play a critical role in innate immunity by directly disrupting microbial membranes and modulating host immune responses [10, 11, 12]. These peptides are widely distributed across bacteria, archaea, and eukaryotes, with amphibians representing one of the richest natural sources identified to date [13, 14, 15, 16, 17]. In particular, numerous peptides have been isolated from frogs, many of which display broad‐spectrum activity by killing or inhibiting the growth of diverse pathogens, including bacteria, fungi, and viruses [18, 19, 20, 21, 22, 23, 24]. Despite their promising bioactivity, the clinical translation of AMPs has been limited by several challenges, including susceptibility to proteolytic degradation, low oral bioavailability, potential cytotoxicity, and high production costs. Nonetheless, ongoing strategies, such as peptide modification [25], cyclization [26, 27], conjugation with nanocarriers [28, 29], and design of synthetic analogs [30], aim to enhance their stability, selectivity, and efficacy.

A crucial step in the optimization of bioactive compounds involves identifying the minimal active region. The resulting simplified core sequence serves as an improved scaffold for rational design modifications. Once the minimal motif is determined, chemists can strategically introduce terminal modifications, non‐natural amino acids (e.g., D‐amino acids), and/or cyclization to enhance biological activity and stability without disrupting the core functional structure. Within this framework, isolating the minimal bioactive core of natural AMPs represents a valuable approach to identify the essential sequence length and structural determinants required for their activity, while potentially reducing synthesis costs and off‐target toxicity [31, 32, 33, 34, 35].

Hs‐1 is an AMP derived from the skin secretion of the Brazilian tree frog *Hypsiboas semilineatus* [36]. The peptide comprises 20 amino acid residues and is predicted to adopt a predominantly α‐helical conformation. Functional studies have shown that Hs‐1 displays selective antimicrobial activity against Gram‐positive bacteria, with minimum inhibitory concentrations (MICs) ranging from 11.7 to 46.6 μM [36]. In addition, Hs‐1 has been reported to inhibit DENV infection by acting directly on the viral particle [37].

In the present study, we evaluated the in vitro antiviral activity of Hs‐1 and a series of its shorter derivatives against a panel of viruses. This investigation was strategically designed to identify the minimal bioactive core within Hs‐1 capable of maintaining, or potentially enhancing, its antiviral efficacy. To achieve this, a set of truncated peptides was designed and characterized, based on the structurally guided selection of the core amphipathic domain derived from a molecular model of Hs‐1 reported in the literature [36]. The peptides were assessed for their secondary structures, antiviral activity, and cytotoxicity in comparison with the full‐length Hs‐1. Finally, the most active compound identified was further modified and thoroughly characterized to evaluate its potential as a lead antiviral agent.

## 2 Results and Discussion

### 2.1 Design, Synthesis of HS1, and Its Truncated Variants

**FIGURE 1:**
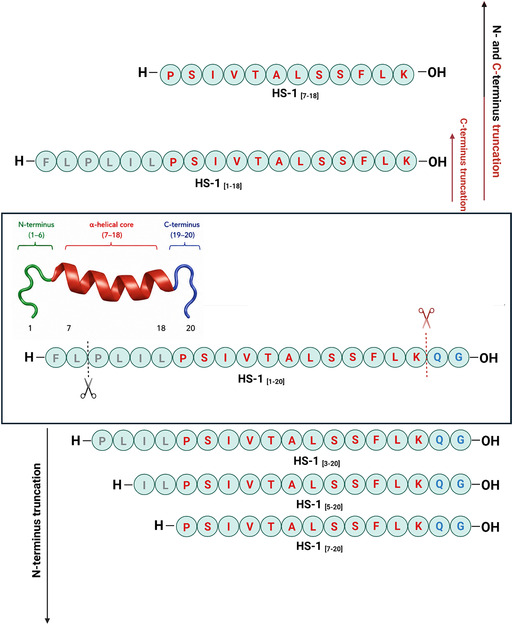
Several truncated derivatives of the Hs‐1 peptide were designed. Each peptide sequence is illustrated as a linear chain of amino acid residues represented by colored circles. The middle box displays the full‐length Hs‐1, which is referred to in the figure as peptide Hs‐1[1–20] for simplicity, and its PEP‐FOLD3‐predicted structure with the N‐terminal region (green), the central α‐helical core (red), and the C‐terminal region (blue). At the top, peptides truncated at both the C‐terminal and N‐terminal are indicated, including Hs‐1[7–18] and Hs‐1[1–18]. The bottom section illustrates the N‐terminal truncation series (Hs‐1[3–20], Hs‐1[5–20], Hs‐1[7–20]) with progressive residue removal from the N‐terminus. Red letters indicate the residues spanning the α‐helical core.

To identify a shorter, more potent analog of Hs‐1 (primary sequence FLPLILPSIVTALSSFLKQG) with enhanced stability, solubility, and antiviral activity, we designed a series of truncated peptides derived from the full‐length sequence. Specifically, based on the molecular model reported in the literature [36], we generated N‐ and C‐terminal deletion variants of Hs‐1 while preserving the putative α‐helical core (spanning residues: PSIVTALSSFLK) (Figure 1).

Table 1 lists the peptides designed and tested. Peptides were synthesized using Fmoc‐based solid‐phase peptide synthesis and purified to homogeneity via RP‐HPLC (see the Materials and Methods section for details). The identity and purity of the peptides were confirmed by LC‐MS analysis (Figures S1–S7).

**TABLE 1:** Primary sequences and monoisotopic theoretical versus experimental molecular weights (M.W.) of the peptides examined.

Peptide name	Sequence	M.W. (theoretical)	M.W. (experimental)
Hs‐1	FLPLILPSIVTALSSFLKQG	2143.26	2142.92
Hs‐1[1–18]	FLPLILPSIVTALSSFLK	1958.18	1957.84
Hs‐1[3–20]	PLILPSIVTALSSFLKQG	1883.10	1883.88
Hs‐1[5–20]	ILPSIVTALSSFLKQG	1672.97	1673.72
Hs‐1[7–20]	PSIVTALSSFLKQG	1446.80	1446.83
Hs‐1[7–18]	PSIVTALSSFLK	1261.72	1261.83

### 2.2 Conformational Analysis by Circular Dichroism (CD) Spectroscopy

**FIGURE 2:**
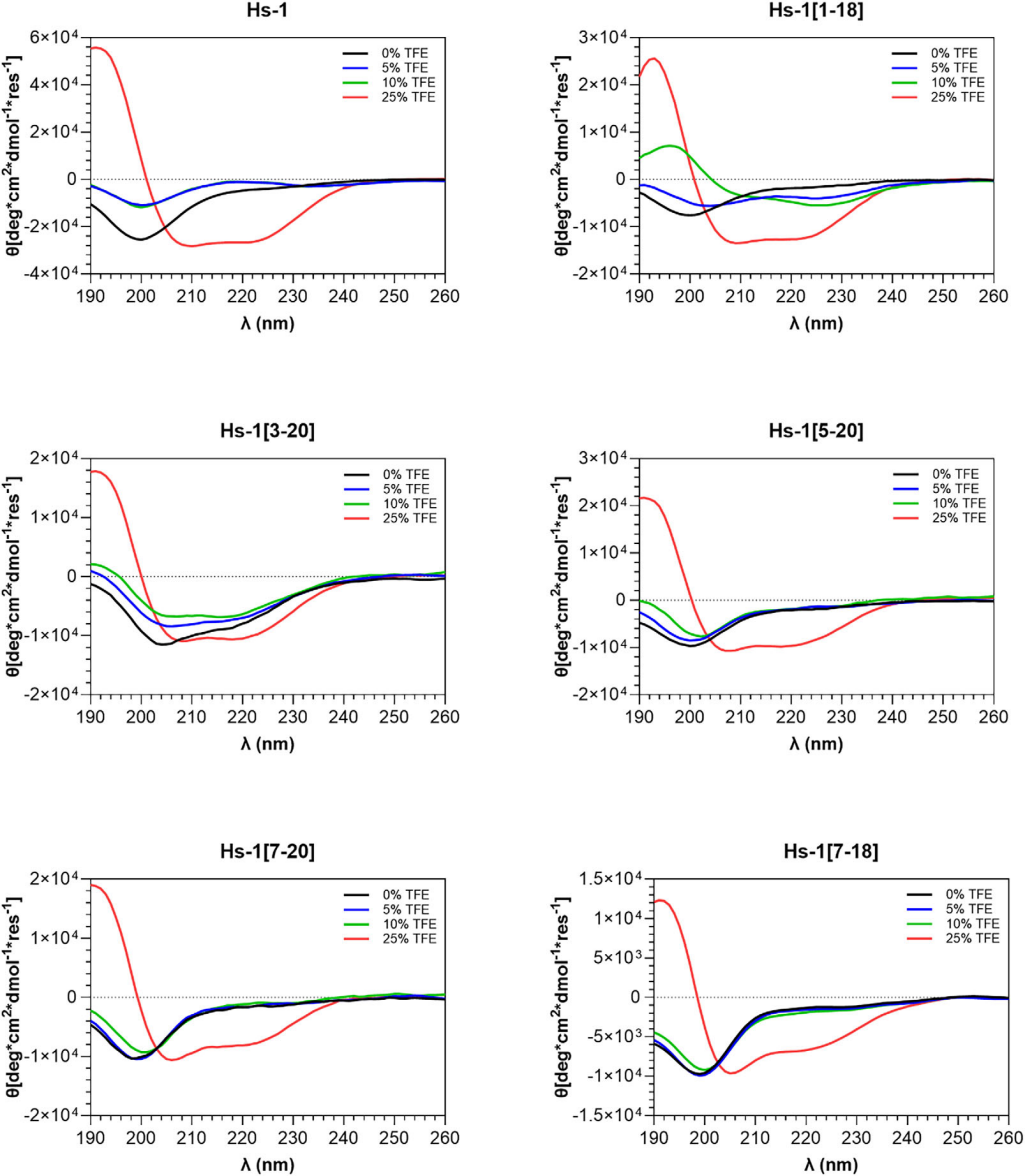
CD spectroscopy of peptides was performed on samples at a concentration of 50 µM in phosphate buffer (5 mM, pH 7.4). Measurements were taken with increasing percentages of TFE to mimic membrane‐like environments. Black lines represent the peptide's CD signal in buffer alone, while signals in 5% (blue lines), 10% (green lines), and 25% TFE (red lines) are also shown for comparison.

To evaluate the structural integrity and conformational behavior of the full‐length Hs‐1 peptide and its truncated derivatives, we performed comparative conformational analysis using CD spectroscopy (Figure 2).

The experimental design involved obtaining and comparing the CD spectra of all variants under two key conditions: (i) aqueous solution (phosphate‐buffered saline, PBS, pH = 7.4), to assess the intrinsic, nonmembrane‐bound state and (ii) a membrane‐mimetic environment induced by trifluoroethanol (TFE), a common agent used to induce AMPs to adopt their membrane‐associated structure (see the Materials and Methods for details). As depicted in Figure 2, the majority of the N‐ and C‐terminal deletion variants exhibited spectra highly similar to that of Hs‐1. In the aqueous solution (Figure 2, black lines), these peptides displayed a single negative ellipticity minimum centered around 198 nm, which is highly characteristic of random‐coil conformations. An exception was the truncated analog, Hs‐1[3–20]. Its spectrum in aqueous solution showed a distinct minimum at approximately 204 nm, accompanied by a shoulder near 220 nm (Figure 2, black line). This spectral signature suggests the presence of mixed conformational states in which an unfolded structure coexists with a partial α‐helical component. The enhanced propensity of Hs‐1[3–20] to spontaneously adopt a helical conformation was further corroborated by the TFE titration analysis. The helical content of Hs‐1[3–20] increased proportionally and rapidly with increasing TFE concentrations, indicating a lower conformational barrier to folding. The other peptides, including the full‐length Hs‐1 peptide, adopted a defined α‐helical conformation (characterized by the double minima at 208 and 222 nm) within the 10%–25% TFE range.

Collectively, these CD results confirm the averaged conformational behavior of the Hs‐1 peptides and their derivatives, showing that they preferentially adopt helical secondary structures in more hydrophobic environments, a crucial requirement for membrane‐active AMPs.

### 2.3 Cytotoxicity Profile and Antiviral Efficacy of Peptides

**FIGURE 3:**
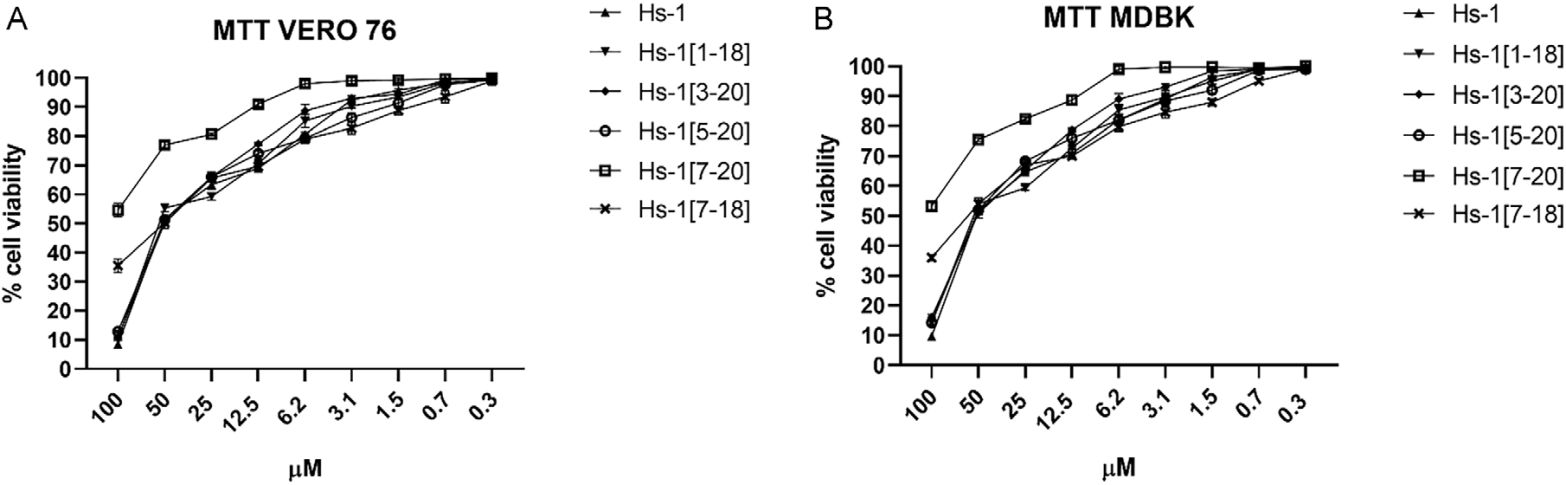
Cytotoxicity evaluation of peptides by MTT assay. Peptides were incubated with (A) VERO 76 and (B) MDBK cell lines for 24 h at concentrations ranging from 0.3 to 100 μM. The percentage (%) of cell viability was calculated relative to the positive control (untreated cells).

To establish the nontoxic concentration range for each peptide, we employed a colorimetric assay that measures mitochondrial metabolic activity in viable cells (see the Materials and Methods section for details). This assay was crucial because it allowed us to identify peptide concentrations that maintain normal cell viability. This step ensures that any antiviral effects observed in successive experiments could be definitively attributed to the specific antiviral activity of the peptides and not to general cytotoxicity. We used two cellular models, VERO 76 and MDBK cells, since these lines were subsequently used for the antiviral assays. As illustrated in Figure 3A,B, the peptide toxicity profiles were consistent across both cell lines tested.

Hs‐1[7–20] reached a CC_50_ at more than 100 μM, whereas the remaining peptides (including the full‐length Hs‐1) were at least twofold more toxic with a CC_50_ around 50 μM (Table 1).

**FIGURE 4:**
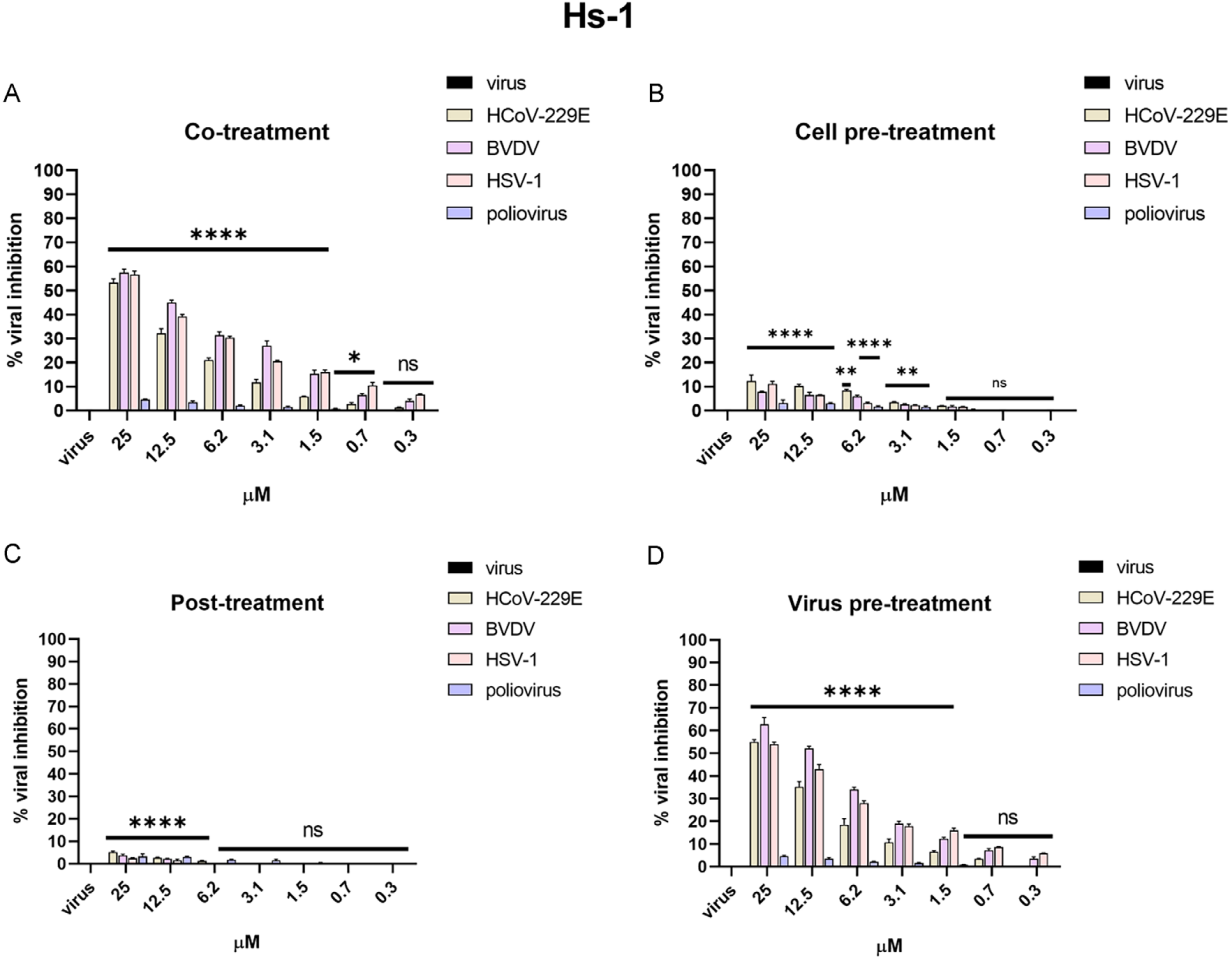
Antiviral activity of Hs‐1 against HCoV‐229E, BVDV, HSV‐1, and poliovirus. Four treatment schemes were employed to assess the antiviral effects of Hs‐1: (A) cotreatment, where the peptide and virus were simultaneously added to cells; (B) cell pretreatment, where cells were preincubated with the peptide before infection; (C) post‐treatment, where infected cells were subsequently exposed to the peptide; and (D) virus pretreatment, where viral particles were preincubated with the peptide before cell inoculation. Viral titers were determined by plaque assay. Data are expressed as mean ± SD from three independent experiments. Statistical significance was determined using one‐way ANOVA followed by Dunnett's post hoc test (****p < 0.0001; **p = 0.0077; *p = 0.034; ns: nonsignificant, vs. virus).

Based on these results, an analysis of the antiviral properties of the full‐length Hs‐1 and all truncated mutants was conducted in the concentration range of 0–25 µM, against a panel of enveloped and nonenveloped viruses. HCoV‐229E, BVDV, and HSV‐1 were used as representative models for class I, II, and III envelope fusion proteins, respectively, while poliovirus was included as a nonenveloped virus. Moreover, each peptide was administered to cells at different stages of viral infection: (a) during infection (cotreatment), (b) before infection (cell pretreatment), (c) after infection (post‐treatment), and (d) following pretreatment of viral particles (virus pretreatment), to have evidence of the mechanism of action of peptides (Figure 4).

Collected data demonstrated that the full‐length Hs‐1 peptide effectively inhibited infection by all tested enveloped viruses, with no observed efficacy against poliovirus. This indicates that its antiviral activity is specifically linked to the presence of the viral envelope. Additionally, Hs‐1 was shown to strongly interfere with the early stages of viral infection, as significant inhibitory effects were observed in both cotreatment and virus pretreatment conditions. Specifically, Hs‐1 exhibited an approximate IC_50_ of 25 μM against HCoV‐229E and HSV‐1 in both assay types. For BVDV, the IC_50_ was 25 μM in the cotreatment assay and 11.2 μM in the pretreatment assay, suggesting an enhanced antiviral effect when the peptide was preincubated with viral particles.

In comparison, the truncated Hs‐1 variants retained significant antiviral activity, achieving 50% inhibition of infection at concentrations up to 25 μM relative to the mock‐infected control group.

Results were summarized in Table 2 and graphs in Figure S7–S11.

**TABLE 2:** CC_50_ values of the tested peptides on VERO 76 and MDBK cell lines. Cytotoxicity was assessed by MTT assay after 24 h of incubation.

Peptide name	CC_50_, μM VERO 76	CC_50_, μM MDBK	IC_50_, μM HCoV‐229E	IC_50_, μM BVDV	IC_50_, μM HSV‐1	IC_50_, μM poliovirus
Hs‐1	51.8	51.8	25	11.2	25	—
Hs‐1[1–18]	55.3	54	12.5	10	12.5	—
Hs‐1[3–20]	50	51	25	25	25	—
Hs‐1[5–20]	51.4	52.1	25	25	25	—
Hs‐1[7–20]	>100	>100	6.25	5	6.25	—
Hs‐1[7–18]	50	54	12.5	12.5	12.5	—

*Note:* IC_50_ values for the truncated peptides against HCoV‐229E, BVDV, HSV‐1, and poliovirus. Data were referred to the virus pretreatment assay. The symbol – refers to the absence of activity. CC_50_ and IC_50_ values were calculated from dose–response curves using GraphPad Prism software.

Hs‐1[3–20] and Hs‐1[5–20] displayed IC_50_ values comparable to the full‐length Hs‐1, equal to 25 μM against HCoV‐229E, BVDV, and HSV‐1. The antiviral activity of Hs‐1[1–18] and Hs‐1[7–18] was enhanced, with IC_50_ values of 12.5 μM against HCoV‐229E and HSV‐1, and about 12.5 μM against BVDV. The most potent truncated peptide was Hs‐1[7–20], which demonstrated significantly lower IC_50_ values of 6.25 μM against HCoV‐229E and HSV‐1, and 5 μM against BVDV. Importantly, none of the peptides, as well as Hs‐1, inhibited poliovirus infectivity.

Overall, the results demonstrated that the amino acid region primarily responsible for the antiviral activity is the central segment spanning residues 7–18 of the Hs‐1 peptide. However, including the final two residues appears to enhance its antiviral efficacy. Consequently, Hs‐1[7–20] emerged as the most effective variant among those tested, both in terms of antiviral activity and safety profile.

### 2.4 Design, Synthesis, and Characterization of the Hs‐1[7–20]mod Peptide

**FIGURE 5:**
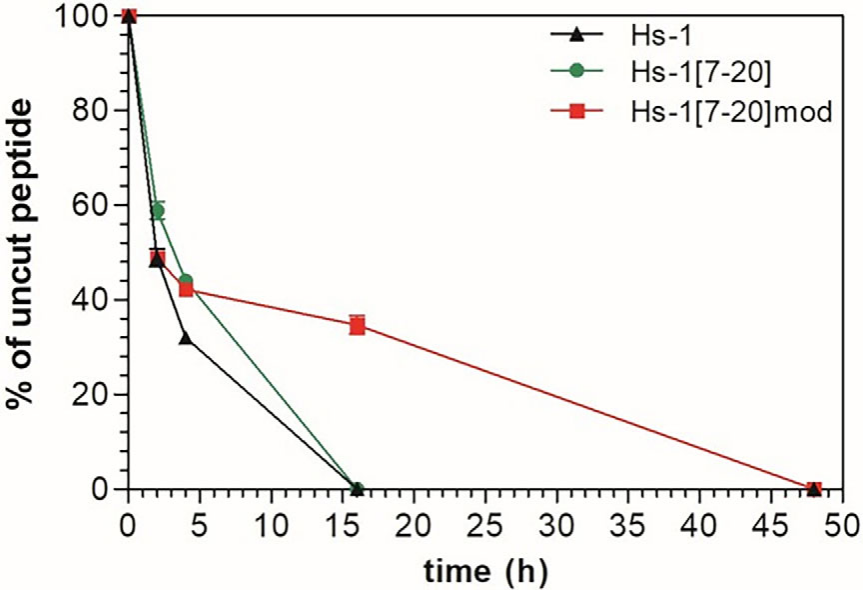
Stability of peptides Hs‐1, HS‐1[7–20], and HS‐1[7–20]mod in 10% (v/v) human serum. This time course plot illustrates the stability of the peptides in the presence of human serum, generated by integrating the corresponding peaks from the RP‐HPLC chromatogram peak integration.

A modified version of Hs‐1[7–20] was designed and synthesized specifically to enhance its stability, following its identification as the most effective variant of the parent compound Hs‐1. The modification involved N‐terminal acetylation and C‐terminal amidation of the Hs‐1[7–20] sequence (see the Materials and Methods section for details). These common terminal modifications are typically employed to improve peptide stability by protecting the termini from enzymatic degradation, thereby increasing the compound's half‐life and biological activity. Following synthesis, the Hs‐1[7–20]mod peptide was purified, and its identity and purity were rigorously confirmed using liquid chromatography‐mass spectrometry (LC‐MS) analysis, as detailed in the Materials and Methods section (Figure S12). Moreover, we assessed the serum stability of the modified peptide, Hs‐1[7–20]mod, compared to the original Hs‐1 and its truncated variant, Hs‐1[7–20]. The degradation of the intact peptides incubated in human serum at 37°C was monitored for 48 h using RP‐HPLC. As shown in Figure 5, both peptides were rapidly degraded, with half‐lives of less than 5 h. However, while Hs‐1 and Hs‐1[7–20] peptides almost completely disappeared after 15 h, no further significant decline occurred for the Hs‐1[7–20]mod peptide over the next 10 h. After 12 h, approximately 40% of the peptide remained in solution.

### 2.5 Cytotoxicity and Antiviral Profile of the Hs‐1[7–20]mod

The newly synthesized Hs‐1[7–20]mod was evaluated for its cytotoxicity under the same experimental conditions used for Hs‐1 and its truncated variants. Like its unmodified precursor, Hs‐1[7–20]mod exhibited a favorable low cytotoxicity profile, showing a CC_50_ > 100 μM (Figure S13). Furthermore, the hemolytic activity of Hs‐1, Hs‐1[7–20], and Hs‐1[7–20]mod was assessed using human erythrocytes (Figure S14). Whereas the parent peptide Hs‐1 induced significant hemolysis at the highest concentrations tested, truncation and terminal modification markedly reduced erythrocyte toxicity. In particular, Hs‐1[7–20]mod exhibited negligible hemolytic activity across the entire concentration range evaluated.

**FIGURE 6:**
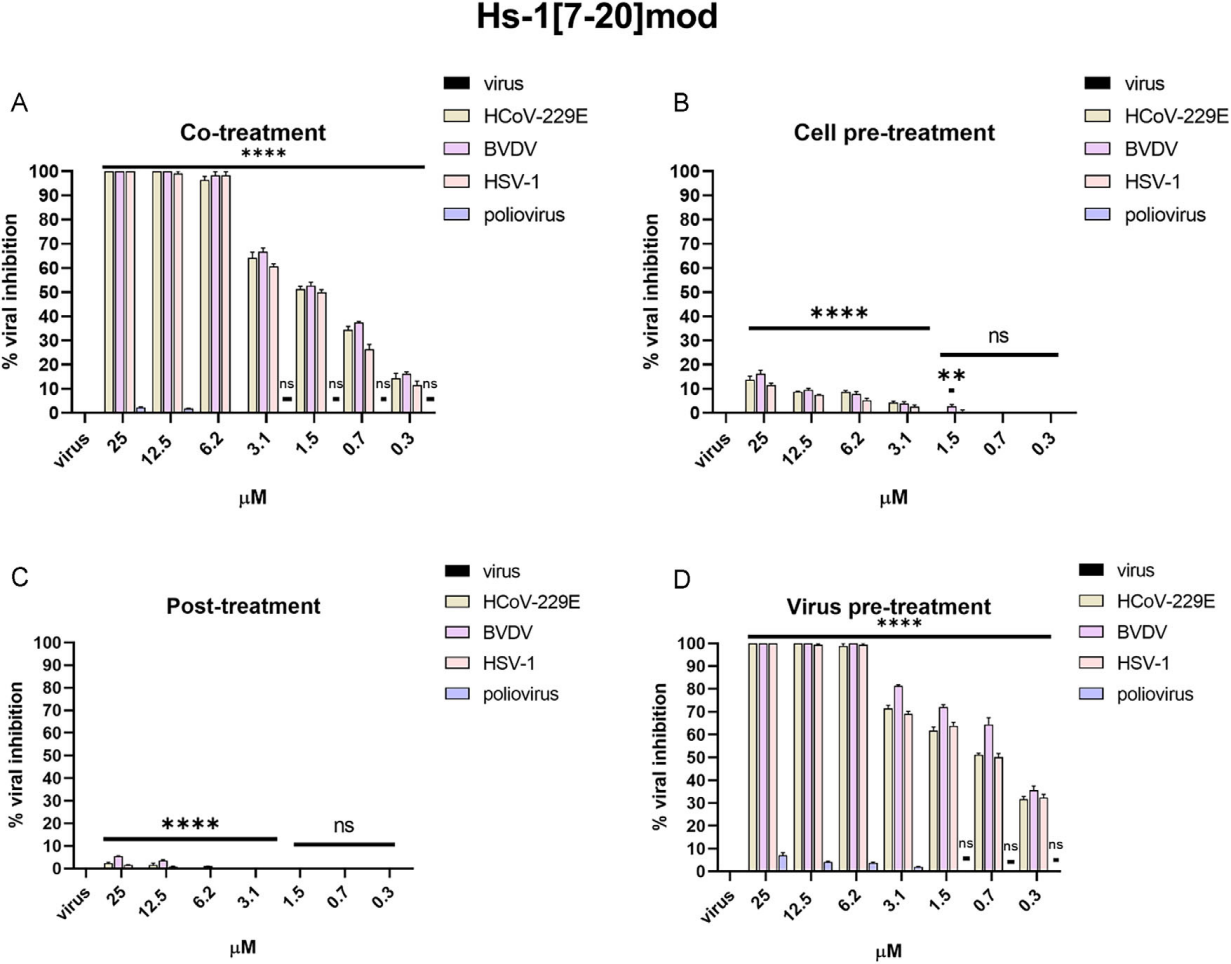
Antiviral activity of Hs‐1[7–20]mod against HCoV‐229E, BVDV, HSV‐1, and poliovirus. Antiviral activity of Hs‐1[7–20]mod under different treatment conditions. Four treatment schemes were tested: (A) cotreatment (peptide and virus added simultaneously), (B) cell pretreatment (cells preincubated with the peptide before infection), (C) post‐treatment (peptide added after infection), and (D) virus pretreatment (virus preincubated with the peptide before cell inoculation). Viral titers were quantified by plaque assay. Data represent the mean ± SD from three independent experiments. Statistical significance was determined by one‐way ANOVA with Dunnett's post hoc test (****p < 0.0001; **p = 0.0016; ns: nonsignificant vs. virus control).

Notably, the incorporated modifications led to a notable enhancement in antiviral activity against all enveloped viruses tested (Figure 6), demonstrating improved efficacy compared to the parental Hs‐1[7–20] peptide (Figure 2).

Specifically, the modified peptide showed an IC_50_ of 1.5 μM in cotreatment across all viruses. Furthermore, in the virus pretreatment setting, its efficacy improved even further, reaching 0.7 μM against HCoV‐229E and HSV‐1, and 0.5 μM against BVDV. Consistent with the putative mechanism of action established for Hs‐1 and its variants, the peptide remained inactive against the nonenveloped poliovirus. Overall, these data clearly demonstrated that the terminal modifications successfully enhanced the antiviral potency of Hs‐1[7–20] without compromising the safety profile of the compound.

### 2.6 Structural Characterization of Hs‐1 and Hs‐1[7–20]mod Peptides by Nuclear Magnetic Resonance (NMR)

To evaluate whether the enhanced activity of the Hs‐1[7–20]mod peptide has a structural basis, we performed a detailed structural characterization of both Hs‐1 and Hs‐1[7–20]mod using NMR.

**FIGURE 7:**
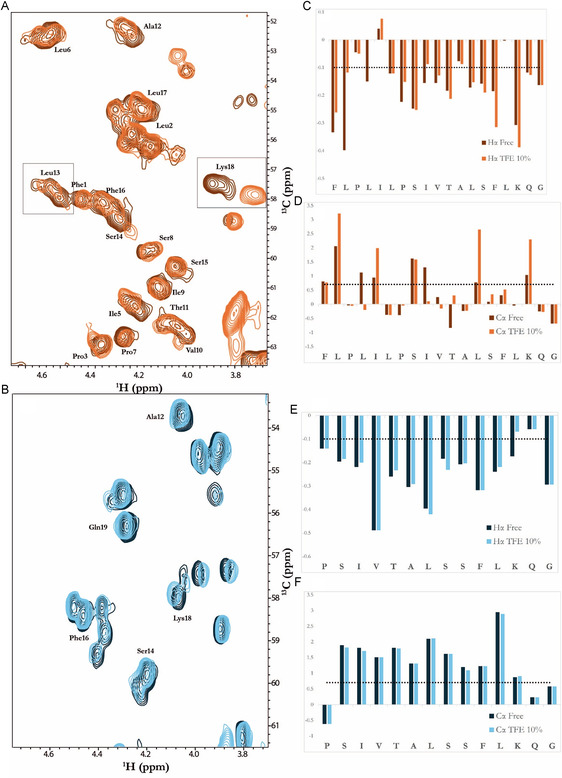
NMR analysis of Hs‐1 and Hs‐1[7–20]mod peptides. (A) Superposition of 1H‐13C HSQC spectra of Hs‐1, in 5 mM phosphate buffer, pH = 7.4 (brown) and in the presence of 10% TFE (v/v) (orange). (B) Superposition of 1H‐13C HSQC spectra of Hs‐1[7–20]mod, in 5 mM phosphate buffer, pH = 7.4 (blue) and after the addition of 10% TFE (v/v) (light blue). (C) Hα secondary shift deviation for Hs‐1 from random‐coil values defined by Kjaergaard and Poulsen [38]. (D) Cα secondary shift deviation for Hs‐1 from random coil values. (E) Hα secondary shift deviation for Hs‐1[7–20]mod from random coil values. (F) Cα secondary shift deviation for Hs‐1[7–20]mod from random coil values. Since a higher TFE concentration, specifically 25% (v/v), was already confirmed by CD analysis to induce a complete helical conformation in all tested peptides, 10% percentage of TFE was specifically chosen for the structural characterization to highlight analogies and differences in the conformational behavior of both peptides.

The NMR structural characterization of both Hs‐1 and Hs‐1[7–20]mod peptides was primarily aimed at evaluating their inherent tendency to adopt an α‐helical conformation. To investigate this behavior, we performed NMR analyses in aqueous phosphate buffer at pH = 7.4 and in solutions containing 10% TFE/phosphate buffer (v/v) (Figure 7).

The sequential assignment of all proton resonances was achieved through 2D ^1^H–^1^H TOCSY (total correlation spectroscopy) and ROESY (rotating frame Overhauser effect spectroscopy) experiments and complemented by 2D ^1^H–^13^C HSQC (heteronuclear single quantum coherence spectroscopy, natural abundance) spectra in both solvent systems. All proton and carbon chemical shifts are reported in Table S1 (Hs‐1) and Table S2 (Hs‐1[7–20]mod) of the Supplemental Materials. Notably, the superposition of HSQC spectra recorded under both experimental conditions for Hs‐1 (Figure 7A) and Hs‐1[7–20]mod (Figure 7B) revealed only minor shifts in both the ^1^H and ^13^C dimensions.

The capability of Hs‐1[1–20] and Hs‐1[7–20]mod to adopt well‐defined structures was evaluated using secondary chemical shift (SCS) analysis. In detail, the peptides Hα and Cα random coil chemical shifts were first predicted using the Kjaegaard et al. approach [38] and then compared with experimental values. The resulting SCS data are shown in Figure 7C–F. According to Weinstock et al. [39], three or more consecutive deviations greater than 0.7 ppm for Cα and −0.1 ppm for Hα indicate a helical arrangement. For Hs‐1, only a few Cα shift residues exceeded the defined threshold, while the Hα SCS analysis (Figure 7C) indicated a slight helical tendency insufficient to drive stable helix formation. These observations are consistent with the CD data, supporting that Hs‐1 exhibits only marginal α‐helical character in aqueous solution and in the presence of 10% TFE (Figure 2). Conversely, the same analysis reports for Hs‐1[7–20]mod has a substantial intrinsic helical propensity in both solvent systems. In fact, the ^1^H–^13^C HSQC spectra of the peptide in aqueous solution and 10% TFE were almost completely superimposable, with the helix‐promoting solvent conditions not altering peak positions. The obtained results are in line with the CD data, which showed that Hs‐1[7–20]mod has a high helical character in aqueous solution, which is further increased in the presence of TFE in a dose‐dependent manner (see Figure S15).

Thus, the SCS analysis strongly suggests that while Hs‐1 displays only weak helical character under membrane‐mimicking conditions, Hs‐1[7–20]mod possesses a stronger intrinsic tendency to form an α‐helix, which likely underlies its enhanced membrane‐interacting capacity.

### 2.7 D Structural Models Prediction of Hs‐1 and Hs‐1[7–20]mod Peptides

**FIGURE 8:**
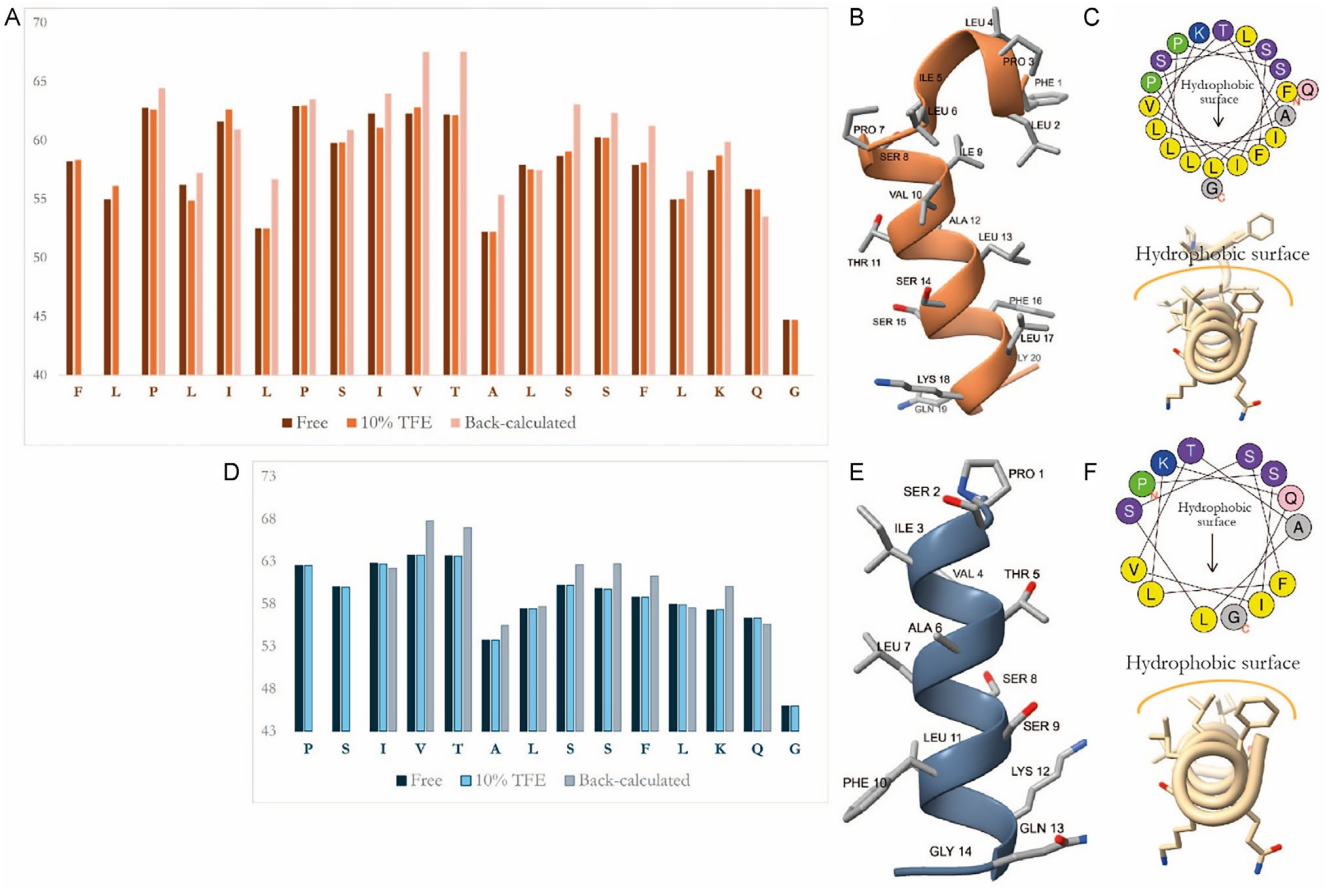
Structural analysis of Hs‐1 and Hs‐1[7–20]mod peptides. (A) Histogram of Cα chemical shifts for Hs‐1 in aqueous buffer (blue), 10% TFE (v/v) (light blue), and back‐calculated from the helical model using PPM_ONE software (gray). (B) Predicted 3D structural motif of Hs‐1. (C) Helix‐wheel diagram of Hs‐1; residues contributing to the hydrophobic surface are underlined in yellow and highlighted in the 3D model. (D) Histogram of Cα chemical shifts for Hs‐1[7–20]mod in aqueous buffer (brown), 10% TFE (v/v) (orange), and back‐calculated from the helical model (pink). (E) Predicted 3D structural motif of Hs‐1[7–20]mod. (F) Helix‐wheel diagram of Hs‐1[7–20]mod; residues forming the hydrophobic surface are underlined in yellow and highlighted in the 3D model.

To further validate our findings, 3D structural predictions were performed using PEP‐FOLD4; the most representative models were selected on the basis of the minimum energy and maximum population and are reported in Figure 8B,E.

Both peptides were predicted to preferentially adopt an α‐helical conformation [39], although this was not observed in aqueous solution. The 3D model of Hs‐1 (Figure 8B) shows a helical segment from Ser8 to Gly20, interrupted by Pro7, which disrupts helix continuity; residues 1–7 adopt a turn‐like structure. The helix‐wheel diagram of Hs‐1 (Figure 8C) highlights its amphipathic character.

In contrast, the 3D model of Hs‐1[7–20]mod (Figure 8E), consistent with its helix‐wheel diagram (Figure 8F), depicts a well‐structured helix with a hydrophobic surface contributed by Leu13, Leu17, and Ile9.

To integrate experimental and computational data, the experimentally determined chemical shifts under both experimental conditions, were compared with shifts back‐calculated from the PEP‐FOLD4 predicted structures using the PPM_ONE software, a well‐established tool for chemical shift prediction. Figure 8A,D shows the comparison obtained for Hs‐1 and Hs‐1[7–20]mod, respectively. The analysis confirms that Hs‐1[7–20]mod exhibits a stronger propensity for the functional conformation, as its experimental chemical shifts align more closely with the predicted values than those of Hs‐1. Notably, the Cα chemical shifts of Leu13, Leu17, and Ile9 closely match the back‐calculated values (Figure 8D). These results confirm that Hs‐1[7–20]mod has a stronger tendency to adopt the biologically active α‐helical conformation capable of interacting with the viral membrane.

## 3 Discussion and Conclusion

Several studies have demonstrated that truncation strategies aimed at isolating the minimal bioactive core can preserve or enhance the antimicrobial potency of natural AMPs, while minimizing production costs and off‐target toxicity. These shortened sequences are designed to retain the key structural features, including overall positive charge, amphipathicity, and critical amino acid residues functionally responsible for membrane disruption [12, 31, 40, 41, 42]. For example, BMAP‐18, a truncated form of the bovine myeloid aAMP BMAP‐27, preserves strong antimicrobial activity while displaying markedly reduced hemolytic effects [43]. Similar success has been achieved with cathelicidins: the 24‐residue derivative GI24, generated from the porcine cathelicidin PMAP‐36, maintains potent broad‐spectrum antimicrobial activity with substantially lower toxicity toward human erythrocytes [32].

Building on this concept, we used structural modeling data to identify the minimal active region of Hs‐1, a 20‐amino‐acid AMP isolated from the Brazilian tree frog *Hypsiboas semilineatus* [36]. Our methodology involved the synthesis of a series of peptide derivatives featuring progressive deletions at both the N‐ and C‐termini, specifically designed to preserve the predicted amphipathic α‐helical core structure (Figure 1) [36]. This targeted approach allowed us to maintain the essential structural determinants for antimicrobial activity while reducing the overall length of the sequence. CD spectroscopy revealed that most peptides tested adopt a predominantly random coil conformation in aqueous solution (Figure 2). An exception was Hs‐1[3–20], which exhibited a mixed conformation with partial α‐helical structure even in aqueous solution. TFE titration experiments confirmed that all peptides could adopt α‐helical conformations in hydrophobic environments, an essential feature for membrane‐active AMPs. Notably, Hs‐1[3–20] required less TFE to achieve this folded state, indicating a lower energetic barrier to adopting the active conformation. Like Hs‐1, all truncated peptides have a similar good safety profile showing a CC_50_ of at least 50 μM with respect to VERO 76 and MDBK cell lines (Figure 3). However, the peptide Hs‐1[7–20], among others, provided the best CC_50_ of 100 μM with respect to both cellular systems. The antiviral activity of these peptides was assessed by determining their IC_50_ values against the three representative enveloped viruses: HCoV‐229E, BVDV, and HSV‐1, in four different experimental schemes (cotreatment, virus pretreatment, cell pretreatment, and post‐treatment). Results obtained generally showed that Hs‐1 (Figure 4) and truncated variants (Figures S7–S11) strongly interfere with the early stages of viral infection, as significant inhibitory effects were observed in both cotreatment and even more in virus pretreatment conditions, and notably, the antiviral assessments against poliovirus, a nonenveloped virus, showed no inhibitory activity across all peptide variants, indicating that the mechanism of action for Hs‐1 and its derivatives is likely target‐specific and primarily effective against enveloped viruses. The time‐of‐addition experiments provide important mechanistic insights into the antiviral action of Hs‐1 and its derivatives. The consistently stronger inhibition observed under virus pretreatment conditions compared with cotreatment assays suggests that direct peptide–virion interactions play a major role in antiviral activity. In virus pretreatment experiments, peptides are allowed to interact with viral particles before cell contact, potentially promoting structural perturbation of the viral envelope and/or masking viral surface proteins required for attachment and entry. In contrast, the reduced efficacy observed during cell pretreatment and post‐treatment assays indicates that these peptides neither significantly alter host cell susceptibility nor interfere with intracellular stages of viral replication.

This hypothesis is further supported by the complete lack of activity against poliovirus, a nonenveloped virus, suggesting that the viral envelope is a primary target of peptide action. Notably, the optimized derivatives Hs‐1[7–20] and Hs‐1[7–20]mod exhibited the greatest improvement specifically under virus pretreatment conditions, indicating that peptide optimization enhanced the ability to interact with and neutralize enveloped viral particles. Their increased amphipathic character, improved α‐helical propensity, and enhanced stability may collectively favor stronger interactions with viral membranes, thereby explaining the superior antiviral efficacy observed for these derivatives. While these findings support direct interactions with enveloped viral particles, the precise molecular basis of such interactions remains to be elucidated. The available data do not allow us to distinguish between membrane perturbation, masking of viral surface proteins, or interactions with specific envelope‐associated components. These possibilities are not mutually exclusive and may collectively contribute to the observed antiviral activity. Further biophysical and ultrastructural studies will be required to clarify this aspect.

Moreover, as summarized in Table 2 (IC_50_ of the peptide detected in virus pretreatment experiments), the results reveal a complex relationship between peptide structure and antiviral potency. Notably, deletion of the C‐terminal residues (Hs‐1[1–18]) (Figure S7) generally resulted in increased antiviral activity compared to the full‐length peptide. Specifically, the IC_50_ against HCoV‐229E and HSV‐1 ranged from 25 μM for Hs‐1 to 12.5 μM for Hs‐1[1–18], indicating that the last two amino acids (residues 19–20) are likely nonessential or even slightly detrimental to activity. Conversely, N‐terminal truncations, such as Hs‐1[3–20] and Hs‐1[5–20], maintained similar IC_50_ (∼25 μM) against HCoV‐229E and HSV‐1 but showed reduced efficacy against BVDV, with IC_50_ around 25 μM compared to 11.2 μM for the parent peptide. These findings suggest that residues at positions 1–4 are not strictly necessary for antiviral activity. Remarkably, truncating the N‐terminus further to generate Hs‐1[7–20] (a 14‐residue peptide) led to a significant two‐ to fourfold enhancement in potency across all tested viruses. This peptide achieved IC_50_ values of approximately 6.25 μM for HCoV‐229E and 5 μM for BVDV, demonstrating both high efficacy and broad‐spectrum antiviral activity. Results imply that the residues within the N‐terminal segment 1–6 may even if they contain elements that stabilize the α‐helical conformation, negatively influence the activity spectrum of the peptides, and their removal results in a more optimized core. The reasons underlying the improved antiviral activity observed after N‐terminal truncation are multifactorial. Since the predicted amphipathic α‐helical core is preserved in Hs‐1[7–20], the enhanced activity cannot simply be attributed to retention of the helical structure. Rather, removal of the N‐terminal segment may promote a more favorable balance between hydrophobicity and cationicity, improve exposure of residues involved in peptide‐virus interactions, and reduce nonproductive interactions with biological membranes. Consistent with this interpretation, the truncated derivatives displayed lower hemolytic activity than the parent peptide, suggesting that optimization of the N‐terminal region not only improved antiviral potency but also enhanced selectivity toward viral targets. Interestingly, the N‐terminal segment removed during the optimization process contains the sequence FLPLI, which shares a high degree of similarity with the FLPII motif previously reported to be associated with immunomodulatory and chemotactic activities in amphibian host‐defense peptides [44]. While deletion of this region markedly enhanced the direct antiviral activity of Hs‐1 derivatives, the possible contribution of the FLPLI‐containing segment to host‐directed immunomodulatory functions was not investigated in the present study. Future studies will be required to determine whether this region contributes to biological activities distinct from the direct antiviral effects described herein.

Further truncation at the C‐terminus to produce Hs‐1[7–18] (12 residues) retained substantial activity. In particular, it showed a twofold improvement against HCoV229E and HSV‐1, with an IC_50_ of 12.5 μM. However, it exhibited a similar potency against BVDV compared to the parent peptide, with an IC_50_ of 12.5 μM. The specificity of Hs‐1[7–18] suggests that the 12‐residue core (residues 7–18) possesses an optimal balance of structural features, such as amphipathicity and charge, for targeting viral particles effectively, making Hs‐1[7–18] a promising candidate for antiviral therapy.

Based on these data, to identify the most effective peptides with the broadest spectrum of activity and the best toxicity profile, we focused our subsequent studies on Hs‐1[7–20]. To optimize the activity of this peptide, we began by introducing chemical modifications at both the N‐ and C‐termini, specifically, acetylation at the N‐terminus and amidation at the C‐terminus, to enhance its half‐life. These terminal modifications did not modify the toxicity profile of the peptide (CC_50_ > 100 μM) (Figure S13) and resulted in a significant improvement in antiviral activity across all tested enveloped viruses (Figure 6). In particular, in a cotreatment setting, the modified peptide, Hs‐1[7–20]mod, exhibited broad‐spectrum efficacy with IC_50_ values in the low‐micromolar range (around 1.5 μM). The efficacy further increased under virus pretreatment conditions, achieving IC_50_ values of 0.7 μM against HCoV‐229E and HSV‐1, and 0.5 μM against BVDV. Finally, NMR spectroscopy (Figures 7 and 8) and serum stability tests (Figure 5) showed that the redesigned peptide is more stable than its precursor peptides and exhibits a slightly increased propensity to adopt α‐helix conformations. These features likely contribute to the observed improvements in antiviral activity.

Although the present study demonstrates the successful optimization of Hs‐1 into a more potent and selective antiviral peptide, several aspects warrant further investigation. Future studies should evaluate the efficacy and safety of Hs‐1[7–20]mod in relevant in vivo models of viral infection to determine its therapeutic potential under physiological conditions. In addition, assessment against a broader spectrum of enveloped viruses, including emerging variants and drug‐resistant strains, will help define the breadth of its antiviral activity. Finally, the development of appropriate formulation and delivery approaches, aimed at enhancing peptide stability, bioavailability, and tissue targeting, may further improve its translational potential as an antiviral therapeutic.

In conclusion, our study clearly demonstrates that the Hs‐1[7–20]mod peptide, despite its smaller size compared to the parent peptide Hs‐1 (14 residues vs. 20 residues, respectively), exhibits favorable safety and serum stability profiles and significantly improved broad‐spectrum antiviral activity. These results position Hs‐1[7–20]mod as an optimized antiviral candidate compared to the parental peptides (Hs‐1 and Hs‐1[7–20]).

## 4 Material and Methods

### 4.1 Synthesis and Analytical Characterization of Peptides

Protected amino acids and coupling agents (HATU, Oxyma) used for peptide synthesis were purchased from Merck (Milan, Italy). Wang and Rink Amide AM resins were obtained from Novabiochem (Milan, Italy). Other reagents employed in the synthesis, including acetonitrile (CH_3_CN), dimethylformamide (DMF), N, N′‐Diisopropylcarbodiimide (DIC), triisopropylsilane (TIS), trifluoroacetic acid (TFA), sym‐collidine, diethyl ether, diisopropylethylamine (DIPEA), and piperidine, were supplied by Merck (Milan, Italy). Peptides were synthesized on solid supports using either Wang resin or Rink Amide AM resin, where indicated, both with an approximate substitution rate of 0.40 mmol/g. Peptide assembly was performed following standard Fmoc chemical protocols, employing Oxyma‐DIC and HATU‐collidine as coupling reagents, in accordance with established methodologies [45], using the Wang resin, with a loading of 0.4 mmol/g for the Hs‐1 peptide and its truncated analogs, to obtain peptides with the carboxyl group at the C‐terminus. Instead, the synthesis of Hs‐1[7–20]mod was performed using Rink Amide AM resin with a loading of 0.4 mmol/g, to obtain the amidated form of the peptide at the C‐terminus. Furthermore, for the same peptide, an acetylation step at the N‐terminus was performed, using a solution of 30% acetic anhydride with 5% DIPEA in DMF (1 mL) for 5 min at room temperature. For the cleavage of all peptides tested in this study, the resin was treated with a mixture of TFA/TIS/H_2_O (90:5:5, v/v/v) for 3 h at room temperature. The crude peptides were then precipitated with cold diethyl ether, dissolved in a water/acetonitrile (70:30, v/v) solution, and lyophilized. Purification was carried out using a WATERS 2545 preparative system (Waters, Milan, Italy) equipped with a WATERS 2489 UV/visible detector. Peptides were purified on a Jupiter C18 column (5 μm, 300 Å, 150 × 21.2 mm [2] ID) using a linear gradient from 5% to 70% CH_3_CN containing 0.1% TFA in water over 15 min at a flow rate of 15 mL/min. Absorbance was monitored at 214 nm. The identity and purity of peptides were confirmed by LC‐MS analysis, performed on an LTQ XL Linear Ion Trap Mass Spectrometer (Thermo Scientific) equipped with a photodiode array (PDA) detector, binary solvent pump with degasser, column heater, and autosampler. LC‐MS analysis utilized an Aeris PEPTIDE XB‐C18 column (3.6 μm, 2.1 × 100 mm) with a linear gradient of CH_3_CN/0.05% TFA in water (from 5% to 80%) over 10 min (min) at a flow rate of 0.2 mL/min. The yields of the synthesized peptides were calculated as the ratio of the actual isolated weight of pure peptide to the theoretical weight based on the initial synthesis scale, resulting in an approximate yield of 80% for each peptide. Purity was assessed by integrating the peak areas at 210 nm, determining the relative purity of each peptide to be up to 95%.

### 4.2 CD Analysis

CD spectra of peptides at 50 μM in 5 mM sodium phosphate buffer (pH 7.4) were recorded from 260 to 190 nm using a JASCO‐705 CD spectropolarimeter (Jasco International Co. Ltd., Tokyo, Japan) at 25°C. Each spectrum was the average of two scans, using a 0.1 cm path length cuvette, with a scan rate of 50 nm min^−^^1^, a 1 s response time, and a 1 nm bandwidth. For 2,2,2‐trifluoroethanol (TFE) titration studies, peptides at 50 μM were titrated with increasing percentages of TFE (5% to 25%). Spectra were acquired as described above and corrected by subtracting the corresponding TFE in buffer spectrum for each specific TFE percentage. Graphs were produced with GraphPad Prism 5.1 (GraphPad Software, San Diego, CA, USA).

### 4.3 Serum Degradation

Peptides’ stability in serum was assessed as previously described [46], with minor modifications. Briefly, peptides were diluted into 10% human serum to a final concentration of 0.5 mg/mL and incubated at 37°C. Aliquots were collected at various time points up to 48 h. For each sample, 30 µL was mixed with 60 µL of 90% ethanol (1:2 ratio) and incubated on ice for 30 min. The mixture was then centrifuged at 12,000 rpm for 10 min. Subsequently, 70 µL of the supernatant was diluted with 210 µL of 0.1% TFA in water (1:3 ratio) and analyzed by RP‐HPLC (Waters e2695 system coupled with a Waters 2998 PDA detector) using an Aeris WIDEPORE C4 column (3.6 µm particle size, 100 × 2.1 mm). A linear gradient from 10% to 60% CH_3_CN containing 0.08% TFA in water was applied over 40 min at a flow rate of 0.2 mL/min. Peptide concentrations were determined from the RP‐HPLC peak areas, normalized to the initial (t0) peak area, which was set as 100%.

### 4.4 Cellsand Viruses

VERO 76 cells (CRL‐1587), derived from *Cercopithecus aethiops* kidney, and Madin‐Darby bovine kidney (MDBK, CCL‐22) cells were maintained in Dulbecco's modified Eagle medium (DMEM) supplemented with 10% heat‐inactivated fetal bovine serum (FBS; Microgem, Naples, Italy), 2 mM L‐glutamine (Microtech, Naples, Italy), and 100 IU/mL of penicillin‐streptomycin (Himedia, Naples, Italy). Cells were incubated at 37°C in a humidified atmosphere containing 5% CO_2_. Human CoV 229E (HCoV‐229E; VR‐740), herpes simplex virus type 1 (HSV‐1; strain SC16), and poliovirus (Sb‐1; VR‐1562) were propagated in VERO 76 monolayers, whereas bovine viral diarrhea virus (BVDV; VR‐534) was cultured in MDBK cells. For viral propagation, confluent cell monolayers were infected with the respective virus at a multiplicity of infection (MOI) of 0.01 and incubated at 37°C until the appearance of virus‐induced cytopathic effect (CPE). Following extensive CPE, the infected cultures were subjected to a single freeze–thaw cycle to release intracellular virions, and the supernatants were clarified by centrifugation at 3000 × g for 10 min. Viral stocks were aliquoted and stored at −80°C until use. All cell lines and viral strains were obtained from the American Type Culture Collection (ATCC, Manassas, VA, USA).

### 4.5 Cytotoxicity Assay

The cytotoxic effects of the tested peptides were evaluated in VERO 76 and MDBK cells using the 3‐(4,5‐dimethylthiazol‐2‐yl)−2,5‐diphenyltetrazolium bromide (MTT) colorimetric assay (Sigma–Aldrich, St. Louis, MO, USA). Cells were seeded in 96‐well plates at a density of 2 × 10^4^ cells per well and incubated overnight at 37°C in a humidified atmosphere containing 5% CO_2_. The next day, cells were exposed to serial dilutions of the peptides for 24 h. Following treatment, MTT solution (0.5 mg/mL) was added to each well and incubated for 3 h to allow formazan formation. The medium was then removed, and the resulting formazan crystals were dissolved in 0.5% dimethyl sulfoxide (DMSO). Absorbance was recorded at 570 nm using a TECAN M‐200 microplate reader (Tecan, Männedorf, Switzerland). Cell viability was expressed as a percentage of untreated control cells according to the following equation:

$$\text{Cell viability }\left(\%\right)=\frac{A_{570\;(\text{treated})}}{A_{570\;(\text{control})}}\times 100$$

### 4.6 Hemolysis Assay

Hemolytic activity was evaluated using fresh human erythrocytes obtained from healthy anonymous donors. Whole blood was centrifuged, and erythrocytes were collected and washed three times with a 150 mM NaCl solution. The cells were then diluted 1:50 in phosphate‐buffered saline (PBS, pH 7.4), and 190 μL of the erythrocyte suspension was dispensed into each well of a 96‐well plate. Hs‐1, Hs‐1[7–20], and Hs‐1[7–20]mod were tested at concentrations ranging from 0.3 to 100 μM. Untreated erythrocytes and erythrocytes treated with 1% Triton X‐100 were used as negative (0% hemolysis) and positive (100% hemolysis) controls, respectively. After incubation for 1 h at 37°C under gentle orbital shaking, the plates were centrifuged at 500 × g for 5 min. Aliquots of the supernatant were transferred to a new plate, and hemoglobin release was quantified by measuring absorbance at 540 nm using a microplate reader.

Hemolytic activity was calculated according to the following equation:

$$\text{Hemolysis }\left(\%\right)=\frac{A_{540\;(\text{treated})}-A_{540\;(\text{negative})}}{A_{540\;(\text{positive})}-A_{540\;(\text{negative})}}\times 100$$

### 4.7 Antiviral Assays

The antiviral effects of the peptides were evaluated in VERO 76 and MDBK cells following established protocols [23, 47, 48]. To investigate the stage of the viral replication cycle affected, several time‐of‐addition assays were performed: (a) Cotreatment: virus and peptide (at noncytotoxic concentrations) were added simultaneously to cell monolayers for 1 h at 37°C, after which the inoculum was removed, cells were washed, and fresh maintenance medium containing carboxymethylcellulose was added; (b) virus pretreatment: the virus was preincubated with peptide for 1 h at 37°C, then diluted and used to infect cell monolayers for 1 h, followed by removal of the inoculum and addition of fresh medium; (c) cell pretreatment: cells were incubated with peptide for 1 h at 37°C, washed, and then infected with virus for 1 h, after which the inoculum was removed and fresh medium added; and (d) post‐treatment: cells were infected with virus for 1 h, washed, and then peptide was added for an additional hour before replacing with fresh medium. Appropriate positive and negative controls were included in all antiviral assays to verify assay performance, reproducibility, and the reliability of the experimental results.

Following incubation (duration depending on the virus), viral replication was quantified by plaque assay or tissue culture infectious dose (TCID_50_) in the appropriate cell line. The 50% inhibitory concentration (IC_50_) was determined by nonlinear regression of the dose–response curve using GraphPad Prism.

### 4.8 Statistics

All experiments were performed in at least three independent replicates. Data are presented as mean ± standard deviation (SD). Statistical comparisons between groups were conducted using one‐way analysis of variance (ANOVA) followed by Dunnett's multiple comparison test, using GraphPad Prism 8.1.2 (GraphPad Software, San Diego, California, United States). Differences were considered statistically significant at *p* < 0.05.

### 4.9 NMR Measurements and Chemical Shift Analysis

NMR experiments were acquired by a Bruker AVII HD 600 MHz spectrometer equipped with a triple‐resonance Prodigy N2 cryoprobe at 298 K. Hs1‐(1–20) and Hs1‐(7–20) peptide samples were prepared in aqueous solution and TFE 10% v/v in 5.0 mM sodium phosphate buffer, pH 7.4, reaching the final concentration of 0.5 mM for each peptide. The final volume was 200 µL + 10% D_2_O in a 3 mm tube. For the sequence assignment, the 2D [^1^H‐^1^H] TOCSY [49] and 2D [^1^H‐^1^H] ROESY [50] data were acquired using 64 scans per t1 increment, a spectral width (SW) of 6001.30 Hz along both t1 and t2, 2048 × 256 complex points in t2 and t1, and a relaxation delay of 3.0 s. Natural‐abundance 2D [^1^H–^13^C] HSQC spectra were acquired with 400 scans per t1 increment, SW of 5130.94 Hz along t1 and 6001.30 Hz along t2, 1024 × 180 complex points in t2 and t1, respectively, and a relaxation delay of 1.0 s. The 2D constant time (CT) [1H–13C] HSQC pulse sequence was optimized as reported in a previous publication [51]. 2D CT [^1^H–^13^C] HSQC were acquired using a heteronuclear coupling constant (J_XH_) of 145 Hz, CT period of 26.6 ms, and shaped pulses for all 180° pulses on the f2 channel with decoupling during f1 acquisition. Two‐dimensional CT [^1^H–^13^C] HSQC spectra were processed with a square cosine window function and zero filled to a final matrix of 4096 × 4096 before Fourier transform and baseline correction. All 2D spectra were processed using CARA software. Cα and Hα secondary shift analysis was performed by random coil values defined by Kjaergaard [38].

### 4.10 Computational 3D Structural Models of HS1‐(1–20) and HS1‐(7–20) Peptides and Chemical Shift Prediction

The model's structural ensemble of Hs‐1[1–20] and Hs‐1[7–20] was obtained using the PEP‐FOLD4 server [52]. For each peptide, starting from the amino acid sequence, the software generated 100 models, and the five conformations with the lowest energy values were selected. PPM_ONE software was applied for the chemical shift prediction, starting from the most representative model calculated by PEP‐FOLD4. All 3D models were visualized and analyzed using ChimeraX software, and the HELIQUEST server was utilized for generating helical wheel diagrams.

## Conflicts of Interest

M.G., A.D.F., C.Z., A.C., and R.G. disclose equity ownership in Matidia Pharma S.R.L. (antiviral *Dimocarpus longan* extract development), C.Z., A.C., and R.G. disclose equity ownership in MicroNature S.R.L. (antiviral chestnut shell extract development). The current research evaluates a de novo synthesized antimicrobial peptide using standard, nonproprietary basic virology and bacteriology testing methods. Because these common methodologies represent the general academic expertise of the authors and involve no proprietary technology or commercial overlap with Matidia Pharma S.R.L. or MicroNature S.R.L., the authors certify that no financial or commercial conflict of interest exists.

## Supporting information

The authors have cited additional references within the Supporting Information [30, 31].

## Data Availability

The data that support the findings of this study are available from the corresponding author upon reasonable request.
