# Supplementary material for: Identification and Optimization of a Truncated Hs‐1‐Derived Antimicrobial Peptide for Enhanced Broad‐Spectrum Antiviral Activity
Source: ChemMedChem. 2026 Jul 8;21(13):e70380. doi: 10.1002/cmdc.70380 (PMC13346337; doi:10.1002/cmdc.70380)
Supplement: Supplementary file 1 — The authors have cited additional references within the Supporting Information [30, 31]. [file CMDC-21-e70380-s001.pdf]

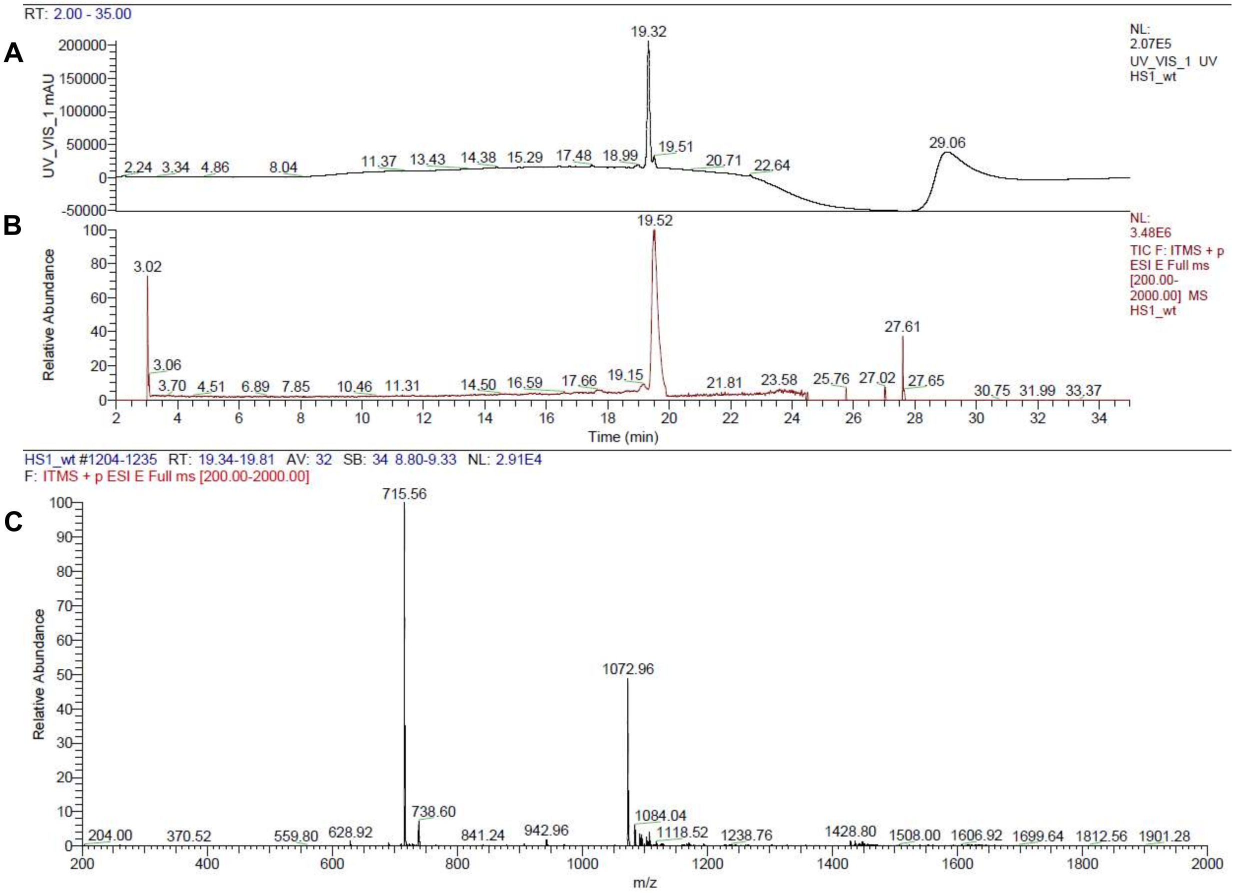

Figure S1. HPLC (A-B) and MS (C) profiles of the purified HS-1. The target peptide exhibited a retention time (tR) of approximately 19.3 min in the HPLC chromatograms (A-B). The MS analysis (C) confirmed the expected molecular weight for HS-1[1-20], with observed m/z values of 1072.96 [M+H]+ and 715.56 [M+2H]2+.

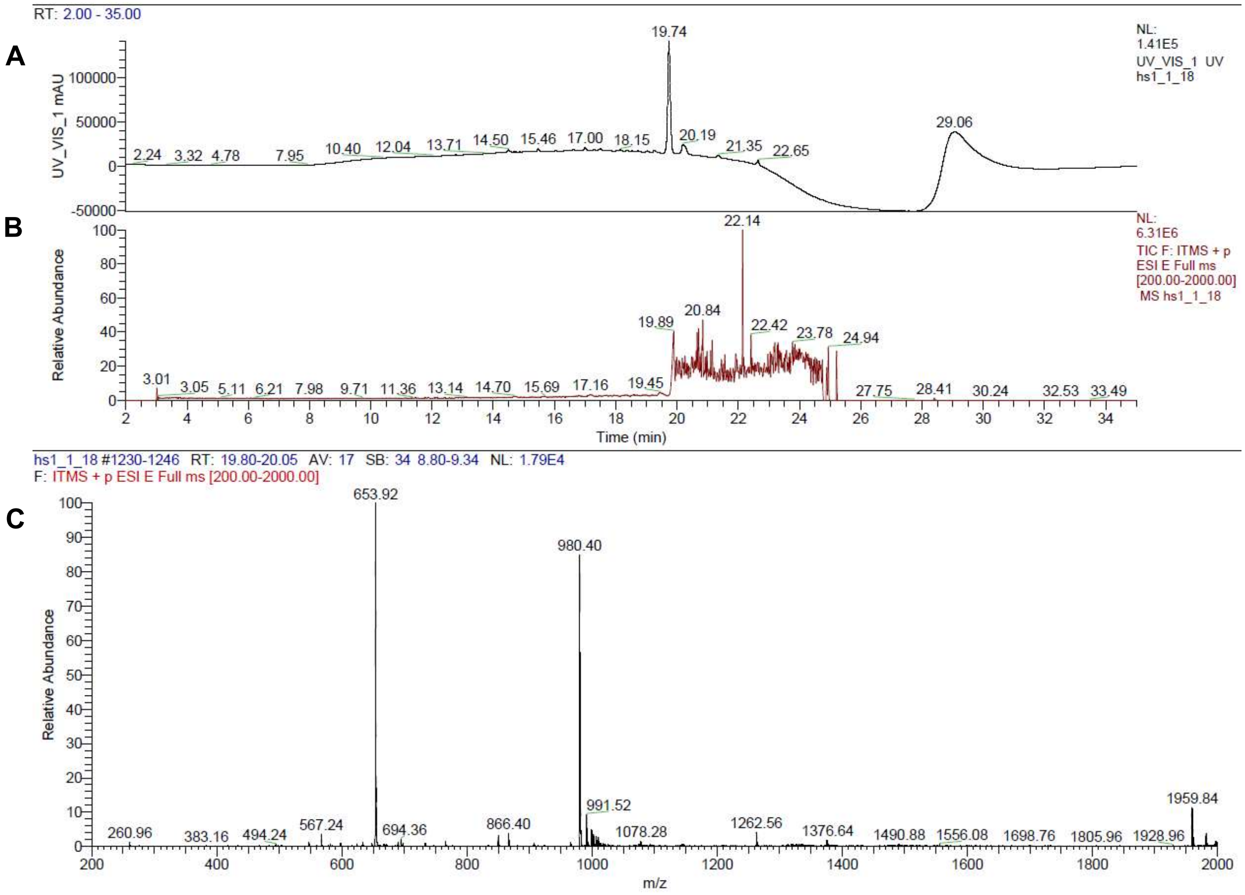

Figure S2. HPLC (A-B) and MS (C) profiles of the purified HS-1[1-18]. The target peptide exhibited a retention time (tR) of approximately 19.7 min in the HPLC chromatograms (A-B). The MS analysis (C) confirmed the expected molecular weight for HS-1[1-18], with observed m/z values of 1959.84 [M+H]+, 980.40 [M+2H]2+, and 653.92 [M+3H]3+.

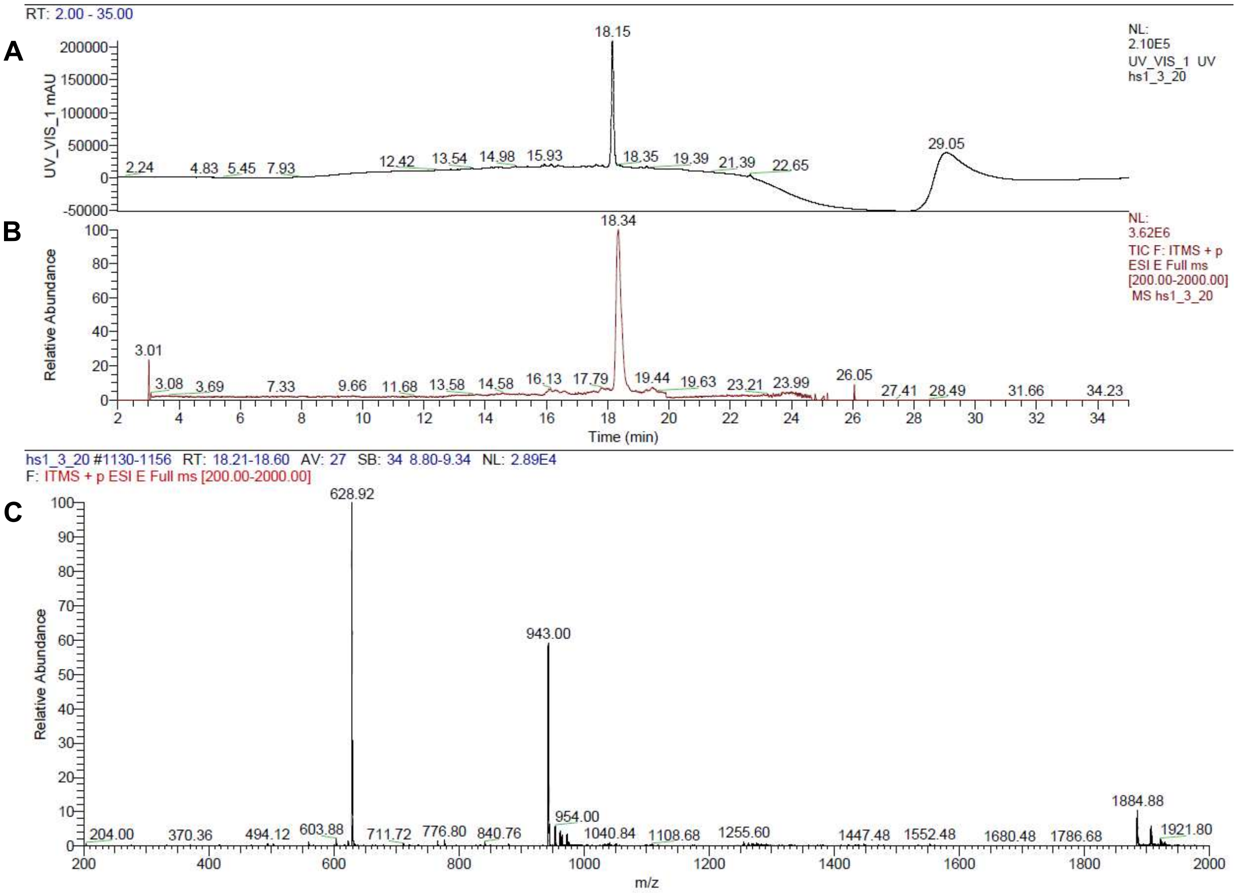

Figure S3. HPLC (A-B) and MS (C) profiles of the purified HS-1[3-20]. The target peptide exhibited a retention time (tR) of approximately 18.2 min in the HPLC chromatograms (A-B). The MS analysis (C) confirmed the expected molecular weight for HS-1[3-20], with observed m/z values of 1884.88 [M+H]+, 943.00 [M+2H]2+, and 628.92 [M+3H]3+.

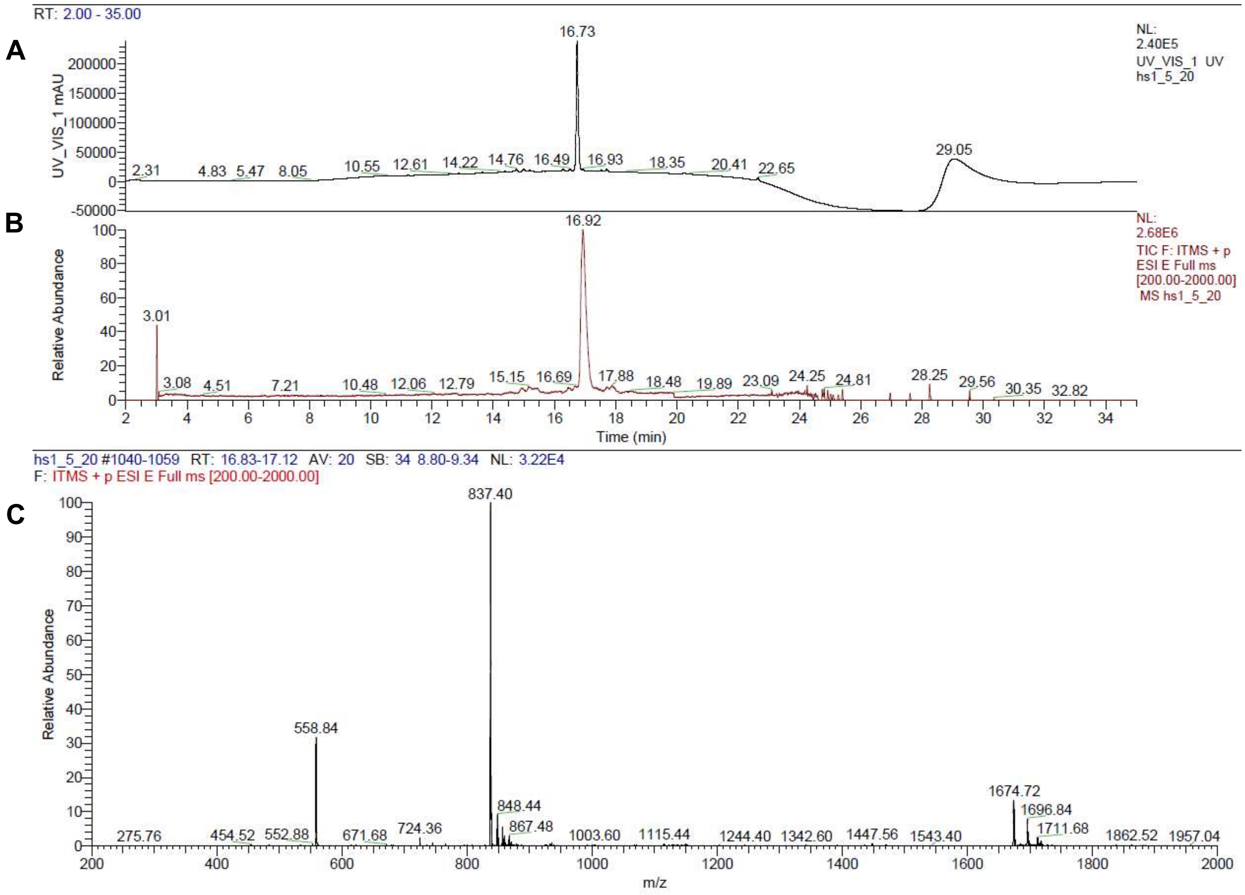

Figure S4. HPLC (A-B) and MS (C) profiles of the purified HS-1[5-20]. The target peptide exhibited a retention time (tR) of approximately 16.7 min in the HPLC chromatograms (A-B). The MS analysis (C) confirmed the expected molecular weight for HS-1[5-20], with observed m/z values of 1674.7 [M+H]+, 837.40 [M+2H]2+, and 558.84 [M+3H]3+.

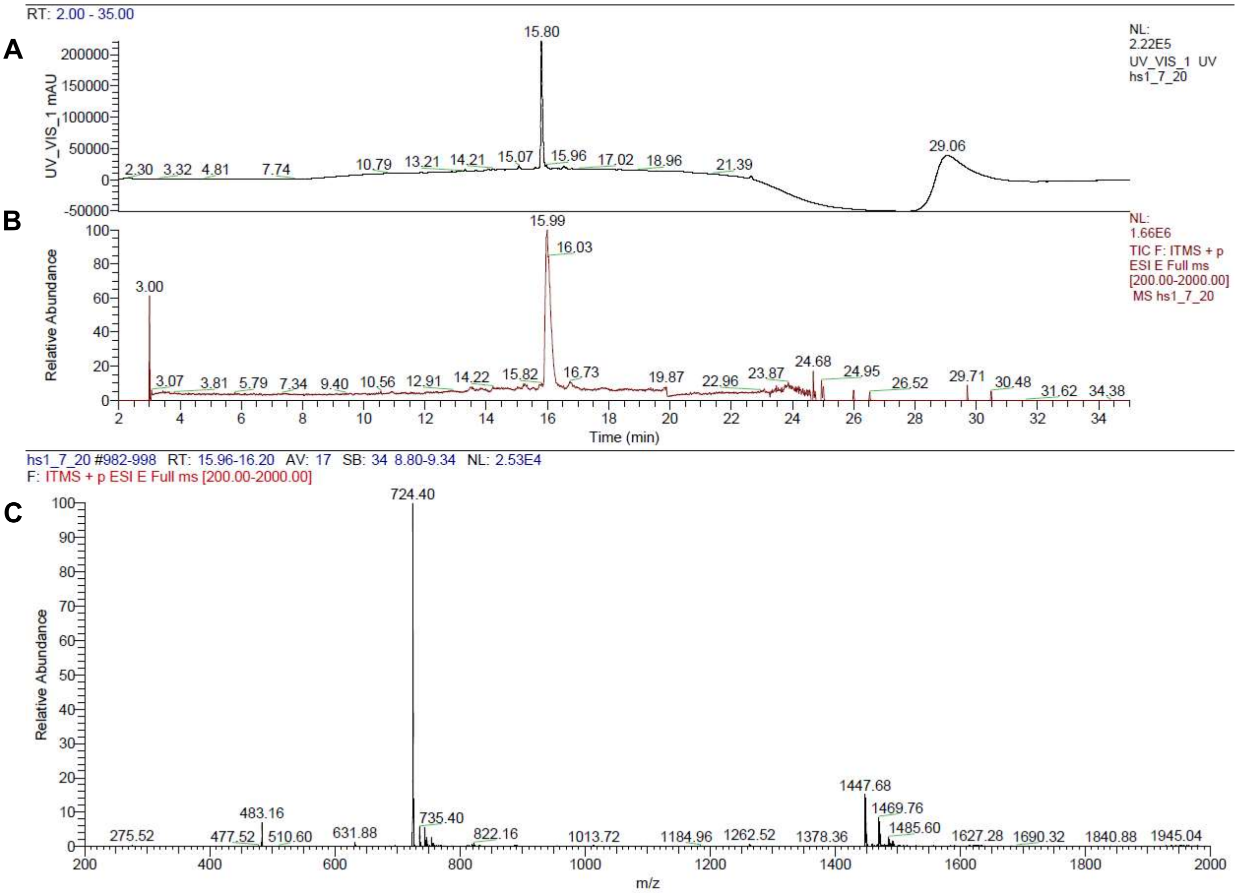

Figure S5. HPLC (A-B) and MS (C) profiles of the purified HS-1[7-20]. The target peptide exhibited a retention time (tR) of approximately 15.8 min in the HPLC chromatograms (A-B). The MS analysis (C) confirmed the expected molecular weight for HS-1[7-20], with observed m/z values of 1447.68 [M+H]+ and 724.40 [M+2H]2+.

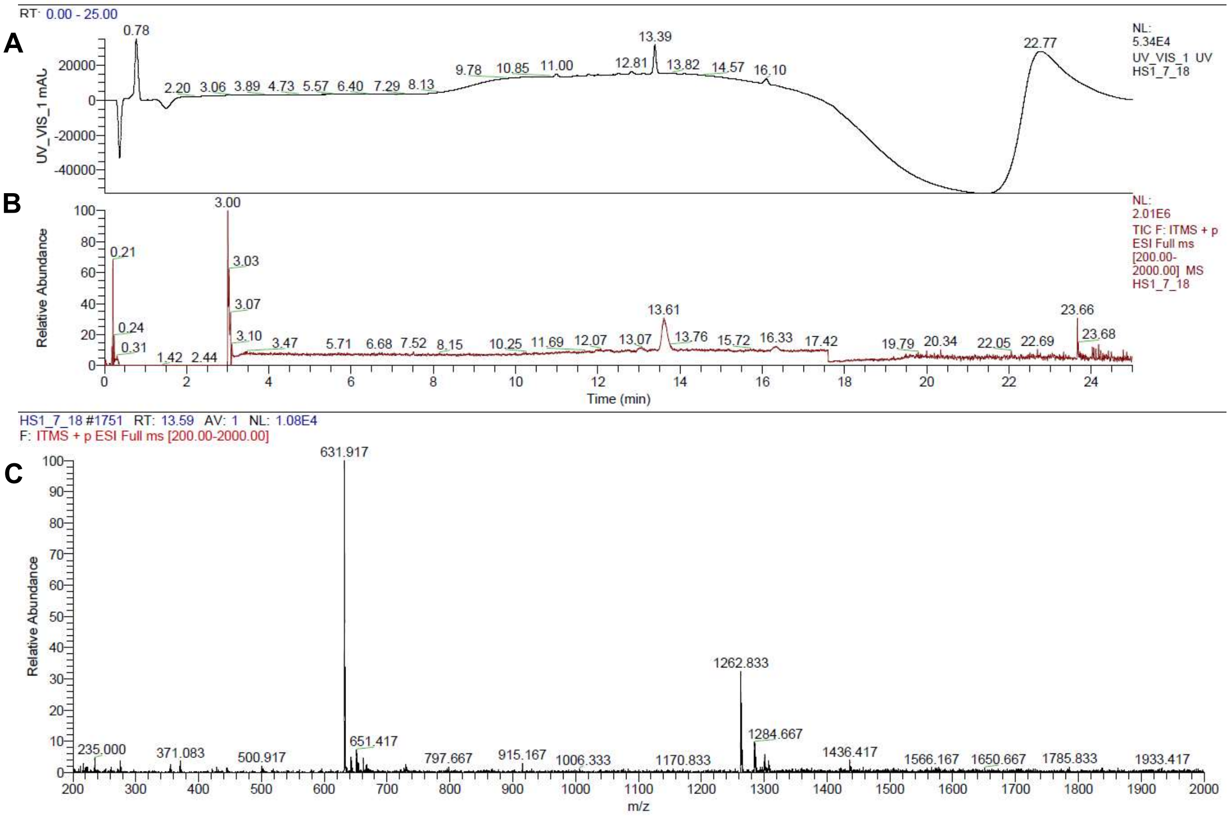

Figure S6. HPLC (A-B) and MS (C) profiles of the purified HS-1[7-18]. The target peptide exhibited a retention time (tR) of approximately 13.4 min in the HPLC chromatograms (A-B). The MS analysis (C) confirmed the expected molecular weight for HS-1[7-18], with observed m/z values of 1262.83 [M+H]+ and 631.92 [M+2H]2+.

Hs-1[1-18]

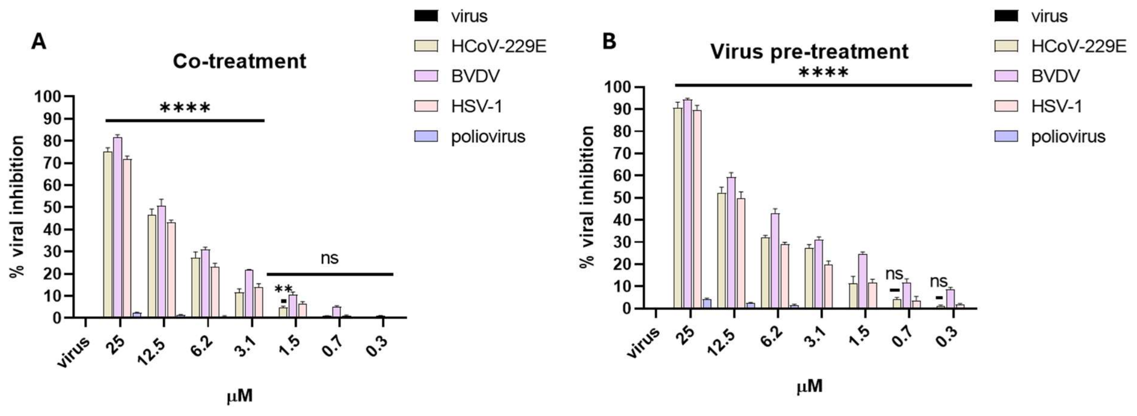

Figure S7. Antiviral activity of Hs-1[1-18] against HCoV-229E, BVDV, HSV-1, and poliovirus. Two treatment conditions were evaluated: (A) co-treatment, in which the peptide and virus were added to the cells simultaneously, and (B) virus pre-treatment, in which viral particles were pre-incubated with the peptide before cell inoculation. Viral titers were quantified by plaque assay. Data are presented as mean ± SD from three independent experiments. Statistical significance was assessed using one-way ANOVA followed by Dunnett's post hoc test (****p <0.0001; **p=0.0016; ns:non-signigicant versus virus control).

Hs-1[3-20]

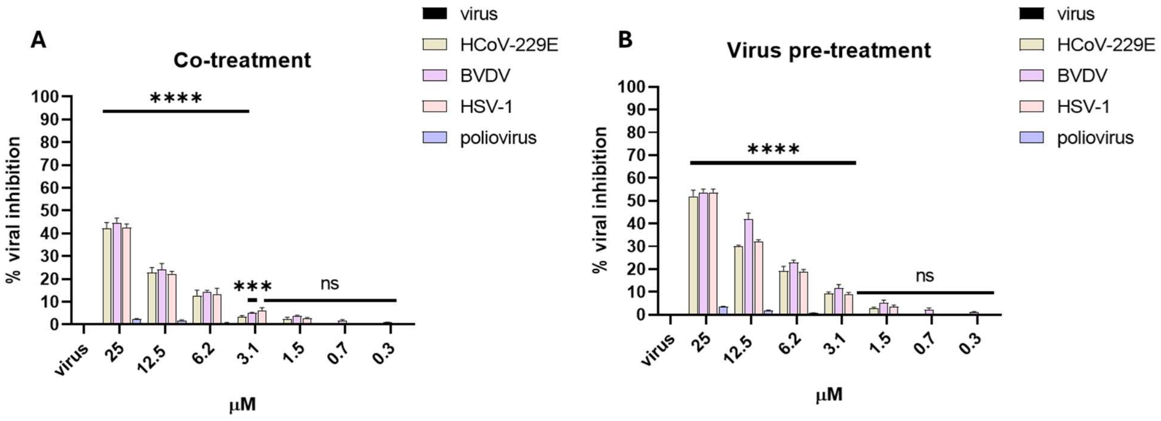

Figure S8. Antiviral activity of Hs-1[3-20] against HCoV-229E, BVDV, HSV-1, and poliovirus. Two treatment conditions were evaluated: (A) co-treatment, in which the peptide and virus were added to the cells simultaneously, and (B) virus pre-treatment, in which viral particles were pre-incubated with the peptide before cell inoculation. Viral titers were quantified by plaque assay. Data are presented as mean ± SD from three independent experiments. Statistical significance was assessed using one-way ANOVA followed by Dunnett's post hoc test (****p < 0.0001; ***p=0.0005; ns:non-significant versus virus control).

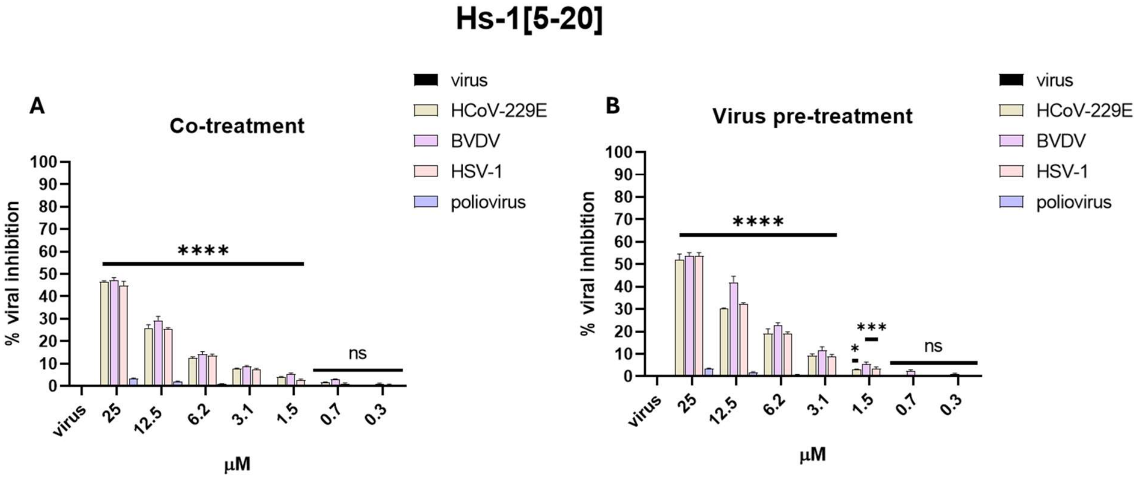

Figure S9. Antiviral activity of Hs-1[5-20] against HCoV-229E, BVDV, HSV-1, and poliovirus. Two treatment conditions were evaluated: (A) co-treatment, in which the peptide and virus were added to the cells simultaneously, and (B) virus pre-treatment, in which viral particles were pre-incubated with the peptide before cell inoculation. Viral titers were quantified by plaque assay. Data are presented as mean ± SD from three independent experiments. Statistical significance was assessed using one-way ANOVA followed by Dunnett's post hoc test (****p <0.0001; ***p=0.0008; *p=0.0406; ns: non-significant versus virus control).

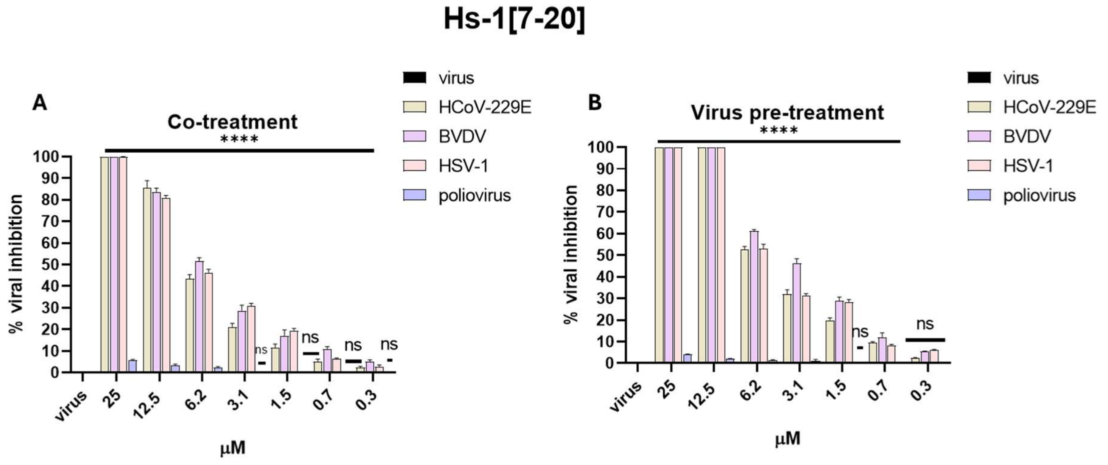

Figure S10. Antiviral activity of Hs-1[7-20] against HCoV-229E, BVDV, HSV-1, and poliovirus. Two treatment conditions were evaluated: (A) co-treatment, in which the peptide and virus were added to the cells simultaneously, and (B) virus pre-treatment, in which viral particles were pre-incubated with the peptide before cell inoculation. Viral titers were quantified by plaque assay. Data are presented as mean ± SD from three independent experiments. Statistical significance was assessed using one-way ANOVA followed by Dunnett's post hoc test (****p <0.0001; ns:non-significant versus virus control).

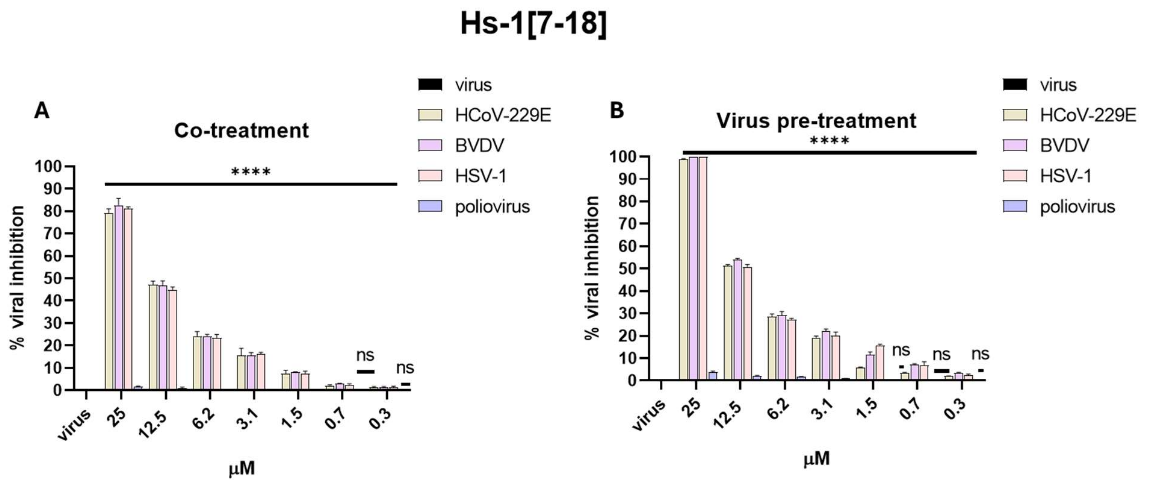

Figure S11. Antiviral activity of Hs-1[7-18] against HCoV-229E, BVDV, HSV-1, and poliovirus. Two treatment conditions were evaluated: (A) co-treatment, in which the peptide and virus were added to the cells simultaneously, and (B) virus pre-treatment, in which viral particles were pre-incubated with the peptide before cell inoculation. Viral titers were quantified by plaque assay. Data are presented as mean ± SD from three independent experiments. Statistical significance was assessed using one-way ANOVA followed by Dunnett's post hoc test (****p <0.0001; ns: non-significant versus virus control).

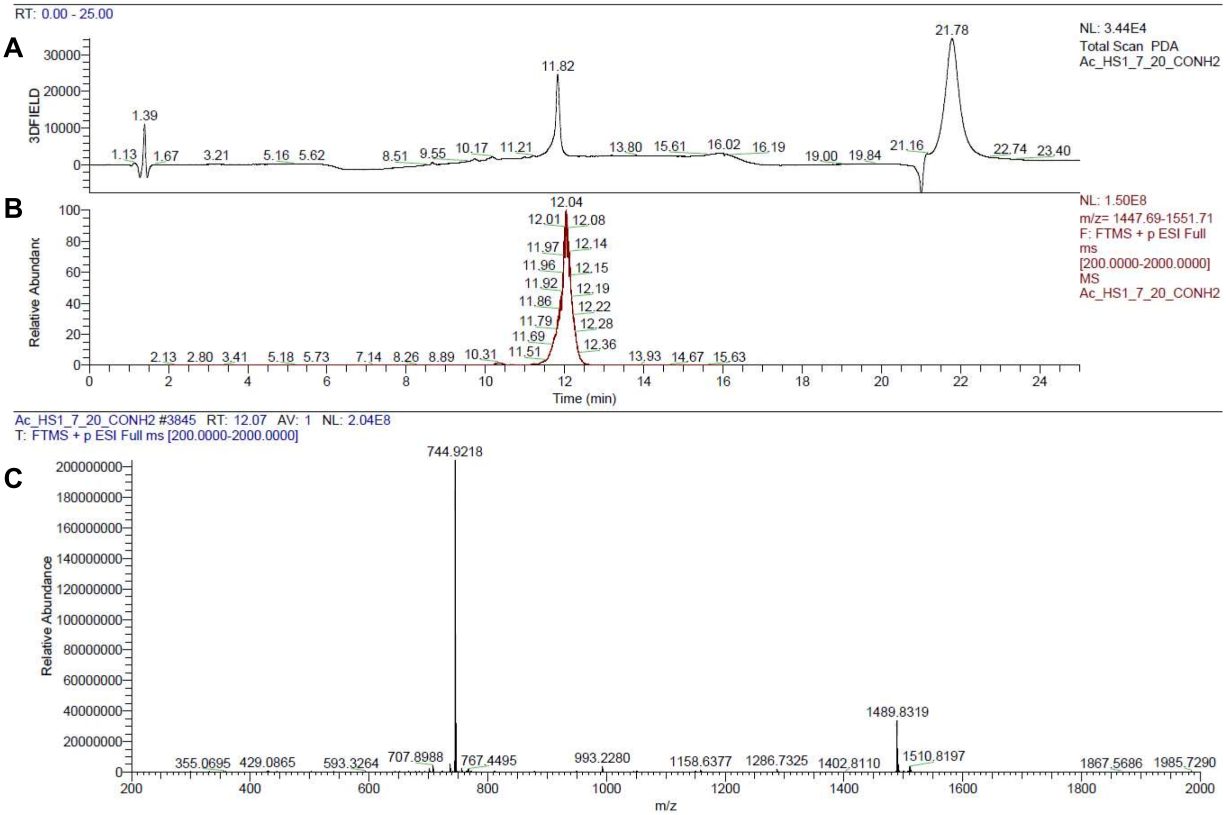

Figure S12. HPLC (A-B) and MS (C) profiles of the purified HS-1[7-20]mod. The target peptide exhibited a retention time (tR) of approximately 11.8 min in the HPLC chromatograms (A-B). The MS analysis (C) confirmed the expected molecular weight for HS-1[7-20]mod, with observed m/z values of 1489.83 [M+H]+ and 744.92 [M+2H]2+.

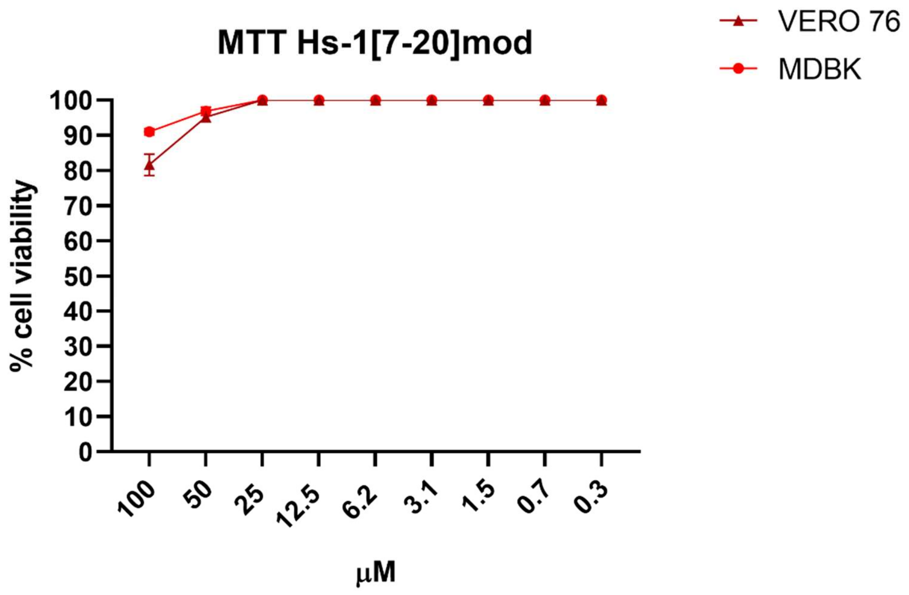

Figure S13. Cytotoxicity of Hs-1[7–20]mod was assessed using the MTT assay. VERO 76 and MDBK cells were incubated with the peptide for 24 h at concentrations ranging from 0.3 to 100 μM. Cell viability (%) was calculated relative to the positive control (untreated cells).

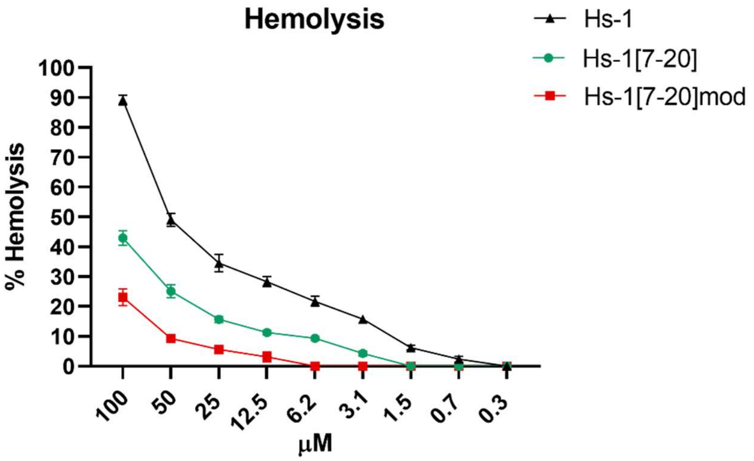

Figure S14. Hemolytic profile of Hs-1, Hs-1[7–20], and Hs-1[7-20]mod. Peptides were tested at concentrations ranging from 0.3 to 100 μM. Hemolysis (%) was calculated relative to the positive control (Triton-treated erythrocytes).

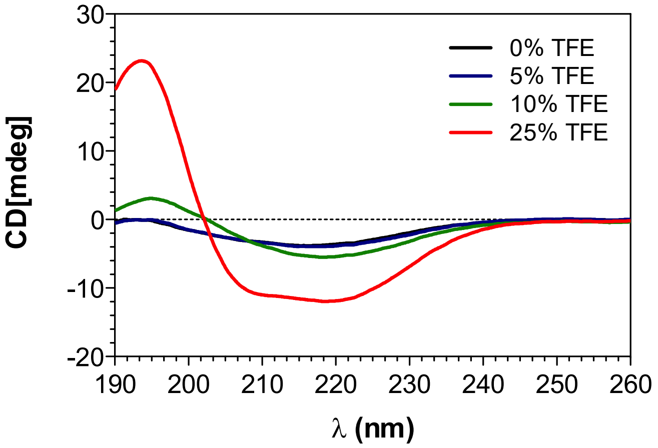

Figure S15. CD spectroscopy of Hs-1[7-20]mod was performed on samples at a concentration of 50 μM in phosphate buffer (5 mM, pH 7.4). Measurements were taken with increasing percentages of TFE to mimic membrane-like environments. Black lines represent the peptide's CD signal in buffer alone, while signals in 5% (blue lines), 10% (green lines), and 25% TFE (red lines) are also shown for comparison.

	F (1)	L (2)	P (3)	L (4)	I (5)	L (6)
NH	8.081	7.882		8.186	7.73	
Hα	4.348 (free and 10%TFE)	4.200(free) 4.182 (10% TFE)	4.354 (free) 4.00 (10%TFE)	4.00(free) 4.088 (10% TFE)	4.228 (free) 4.264 (10% TFE)	4.555(free and 10% TFE)
Hβ	Hβ2 3.026	Hβ2 1.493	Hβ2 2.230	Hβ2 1.483	1.782	Hβ2 1.543
	Hβ3 2.914	Hβ3 1.488	Hβ3 1.837	Hβ3 1.562		Hβ3 1.477
Hγ			Hγ2 1.803		Hγ12 1.123	
			Hγ3 1.951		Hγ13 1.087	
Hδ	Hδ1 7.179	0.821	Hδ2 3.589	0.856	0.828	0.85
	Hδ2 7.123		Hδ3 3.765			
Hε	Hε1 7.237					
	Hε2 7.290					
Cα	58.068(free) 58.106 (10%TFE)	56.147 (free) 54.987 (10% TFE)	62.883 (free) 62.903 (10%TFE)	54.904 (free) 56.225 (10% TFE)	62.652 (free) 61.634 (10% TFE)	52.517 (free) 52.525(10% TFE)
Cβ	39.207	43.235	31.89	42.133	38.593	41.56
Cγ			27.134		Cγ1 27.004	
					Cγ2 17.236	
Cδ		23.251	50.4	23.719	12.343	24.699
	**P (7)**	**S (8)**	**I (9)**	**V (10)**	**T (11)**	**A (12)**
NH		7.996	8.037	8.11	7.746	8.201
Hα	4.274 (free) 4.346 (10%TFE)	4.196 (free) 4.191 (10% TFE)	4.086 (free) 4.154 (10% TFE)	4.051 (free) 4.078 (10%TFE)	4.146(free) 4.117 (10% TFE)	4.276(free) 4.265 (10% TFE)
Hβ	Hβ2 2.206	Hβ2 3.741	1.726	1.681	4.125	1.31
	Hβ3 1.794	Hβ3 3.863				
Hγ	Hγ2 1.925		Hγ12 1.095			
	Hγ3 1.939		Hγ13 1.368			
Hδ	Hδ2 3.634		0.776			
	Hδ3 3.534					
Cα	62.968 (free) 62.628 (10%TFE)	59.686 (free) 59.719 (10% TFE)	61.097 (free) 62.300 (10% TFE)	62.124 (free) 62.532 (10%TFE)	62.176(free) 61.028 (10% TFE)	52.208(free) 52.203(10%TFE)
Cβ	31.859	63.375	38.277	32.476	69.619	18.959

Cγ	27.116		Cγ1 26.956	Cγ1 20.964		
			Cγ2 17.272	Cγ2 20.476		
Cδ	50.301		12.687			
	**L (13)**	**S (14)**	**S (15)**	**F (16)**	**L (17)**	**K (18)**
NH	8.136	8.23	7.908	7.64	8.366	7.975
Hα	4.217 (free) 4.147 (10% TFE)	4.291 (free) 4.259 (10% TFE)	4.061(free) 4.079 (10% TFE)	4.507(free) 4.377 (10%TFE)	4.304(free) 4.301 (10% TFE)	3.957 (free) 3.876 (10% TFE)
Hβ	Hβ2 1.445	Hβ2 3.767	Hβ2 3.773	Hβ2 3.075	Hβ2 1.450	Hβ2 1.634
	Hβ3 1.509	Hβ3 3.921	Hβ3 3.709	Hβ3 2.982	Hβ3 1.525	Hβ3 1.740
Hγ						Hγ2 1.309
						Hγ3 1.347
Hδ	0.842			Hδ1 7.640	0.853	Hδ2 1.435
				Hδ2 7.227		Hδ3 1.476
Hε				Hε1 7.247		
				Hε2 7.277		
Cα	57.995 (free) 56.125(10% TFE)	59.941 (free) 58.671 (10% TFE)	60.26 (free) 60.273 (10% TFE)	58.124 (free) 57.922 (10%TFE)	55.021 (free) 54.972 (10% TFE)	58.732 (free) 57.471 (10% TFE)
Cβ	41.794	63.182	63.732	39.133	42.159	32.681
Cγ						24.506
Cδ	23.796				24.699	26.687

	**Q (19)**	**G (20)**
NH	8.305	8.2

Hα	4.243 (free)<br>4.234 (10%TFE)	3.806 (free and 10%TFE)
Hβ	Hβ2<br>2.040	
	Hβ3<br>1.923	
Hγ	Hγ2<br>2.295	
	Hγ3<br>2.351	
Hδ		
Hε		
Cα	55.830 (free)<br>55.849 (10%TFE)	44.734 (free and 10%TFE)
Cβ	29.185	
Cγ	33.575	
Cδ		

Table S1. Proton and aliphatic carbon chemical shifts (ppm) of Hs-1. Chemical shift values in the presence of 10% TFE are reported for Hα and Cα.

	**P (7)**	**S (8)**	**I (9)**	**V (10)**	**T (11)**	**A (12)**
NH		8.151	7.73	8.068	8.068	7.877
Hα	4.356 (free and 10%TFE)	4.247 (free)<br>4.258 (10% TFE)	4.023 (free)<br>4.042 (10% TFE)	3.719 (free and 10%TFE)	4.070(free)<br>4.096 (10% TFE)	4.049(free)<br>4.061 (10% TFE)
Hβ	Hβ2<br>2.418	Hβ2<br>3.665	1.637	1.681	3.718	1.256
	Hβ3<br>2.326	Hβ3<br>3.82				
Hγ	Hγ2<br>2.033		Hγ12<br>1.051			
	Hγ3<br>1.925		Hγ13<br>1.325			
Hδ	Hδ2<br>3.347		0.776			
	Hδ3<br>3.683					
Cα	62.538 (free and 10%TFE)	60.03 (free)<br>60.00	62.803 (free)<br>62.705 (10% TFE)	63.787 (free and 10%TFE)	60.675(free)<br>63.65 (10% TFE)	53.747 (free and 10%TFE)

		(10% TFE)				
Cβ	31.859	63.638	40.068	32.476	67.365	18.959
Cγ	27.116		Cγ1 26.956	Cγ1 20.964		
			Cγ2 17.272	Cγ2 20.476		
Cδ	50.748		12.687			
	**L (13)**	**S (14)**	**S (15)**	**F (16)**	**L (17)**	**K (18)**
NH	7.759	7.991	7.876	8.077	8.006	8.061
Hα	3.993 (free) 3.969 (10% TFE)	4.265 (free) 4.218 (10% TFE)	4.200 (free) 4.205 (10% TFE)	4.374 (free and 10%TFE)	4.068(free) 4.088 (10% TFE)	4.090 (free) 4.195 (10% TFE)
Hβ	Hβ2 1.445	Hβ2 3.767	Hβ2 3.752	Hβ2 3.126	Hβ2 1.496	Hβ2 2.206
	Hβ3 1.480	Hβ3 3.921	Hβ3 3.825	Hβ3 2.899	Hβ3 1.488	Hβ3 1.997
Hγ						Hγ2 0.874
						Hγ3 0.856
Hδ	0.801			Hδ1 6.778	0.82	Hδ2 1.979
				Hδ2 6.724		Hδ3 1.843
Hε				Hε1 7.447		
				Hε2 7.523		
Cα	57.447 (free) 57.456(10% TFE)	59.824 (free) 60.00 (10% TFE)	59.824 (free) 60.00 (10% TFE)	58.823 (free and 10%TFE)	57.965 (free) 57.9110 (10% TFE)	57.304 (free) 57.343 (10% TFE)
Cβ	41.794	63.182	63.605	39.421	42.159	32.681
Cγ						24.315
Cδ	23.424				24.699	26.687

	Q (19)	G (20)
NH	8.51	8.033
Hα	4.00 (free and 10%TFE)	3.675 (free and 10%TFE)
Hβ	Hβ2 2.027	
	Hβ3 1.909	
Hγ	Hγ2 2.295	
	Hγ3 2.351	
Hδ		
Hε		
Cα	56.334 (free and 10%TFE)	46 (free and 10%TFE)
Cβ	29.185	
Cγ	33.575	
Cδ		

Table S2. Proton and aliphatic carbon chemical shifts (ppm) of Hs-1 [7-20] mod. Chemical shift values in the presence of 10% TFE are reported for Hα and Cα.
